# One-pot two-step catalytic synthesis of 6-amino-2-pyridone-3,5-dicarbonitriles enabling anti-cancer bioactivity†

**DOI:** 10.1039/d2ra03579k

**Published:** 2022-08-24

**Authors:** Lynden G. Nicely, Ruturajsinh M. Vala, Dipti B. Upadhyay, Joaquina Nogales, Celestine Chi, Sourav Banerjee, Hitendra M. Patel

**Affiliations:** Department of Cellular and Systems Medicine, School of Medicine, University of Dundee Dundee DD1 9SY UK s.y.banerjee@dundee.ac.uk; Department of Chemistry, Sardar Patel University Vallabh Vidyanagar 388120 Gujarat India hm_patel@spuvvn.edu; Department of Medical Biochemistry and Microbiology, Uppsala University SE-75123 Uppsala Sweden

## Abstract

We report a one-pot two-step synthesis of a bioactive 6-amino-2-pyridone-3,5-dicarbonitrile derivative using natural product catalysts betaine and guanidine carbonate. Anti-cancer bioactivity was observed in specific molecules within the library of 16 derivatives. Out of the compounds, 5o had the most potent anti-cancer activity against glioblastoma cells and was selected for further study. Compound 5o showed anti-cancer properties against liver, breast, lung cancers as well as primary patient-derived glioblastoma cell lines. Furthermore, 5o in combination with specific clinically relevant small molecule inhibitors induced enhanced cytotoxicity in glioblastoma cells. Through our current work, we establish a promising 6-amino-2-pyridone-3,5-dicarbonitrile based lead compound with anti-cancer activity either on its own or in combination with specific clinically relevant small molecule kinase and proteasome inhibitors.

## Abstract 2

The one-pot synthetic strategy of amino-pyridones with potent anti-cancer activity against breast, brain, liver, and lung cancer cells either alone or in combination with clinically relevant receptor tyrosine kinase and proteasome inhibitors.

## Graphical abstract

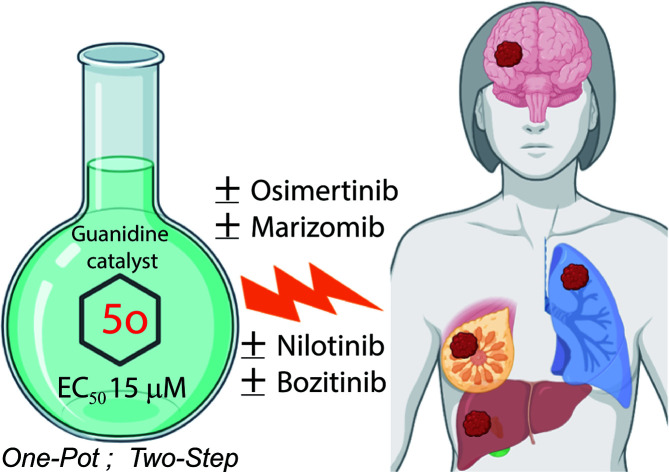

## 1. Introduction

Establishing novel and cost-effective strategies to synthesise unique bioactive chemical scaffolds is essential for future development of cancer therapeutics. For some forms of cancers like glioblastoma (stage IV brain cancer),^1^ there are very limited options for patients and is categorised as a major ‘unmet need’. Various blood–brain-barrier (BBB) penetrant small molecules have either undergone or are currently undergoing clinical evaluations like PI-103,^2^ buparlisib,^3^ abemaciclib,^4^ bozitinib,^5^ marizomib,^6^ nilotinib,^7^ osimertinib^8^ and others. However, maintaining a large and diverse pipeline of novel cancer therapeutics remains the need of the hour.

Previously, we had successfully established a synthetic strategy of 6-amino-2-pyridone-3,5-dicarbonitriles^9^ using piperidine acetate as a catalyst. To further establish a non-hazardous synthetic approach for 6-amino-2-pyridone-3,5-dicarbonitriles, we decided to utilise a natural product catalyst. The main advantages of natural product catalysts are that they require milder reaction conditions and do not contain any heavy metal, making them non-toxic. In our previous work, we synthesised pyrazolo[3,4-*b*]quinolinones using the natural product pyridine-2-carboxylic acid^10^ and spiro-heterocycles by using the natural product betaine based deep-eutectic solvents.^11,12^ Herein, we explored the catalytical activity of pyridine-2-carboxylic acid and betaine for the synthesis of 6-amino-2-pyridone-3,5-dicarbonitriles. We also explored the catalytical activity of guanidine carbonate for the reaction. As a catalyst, guanidine carbonate is used in the methanolysis of triacylglycerols^13^ and synthesis of graphitic carbon nitride.^14^ It is rarely used to catalyse one-pot synthesis; however, it has been used as a substrate in one-pot multicomponent synthesis.^15–17^ We explored the catalytical activity of guanidine carbonate in one-pot synthesis of 6-amino-2-pyridone-3,5-dicarbonitriles. Consequently, we observed anti-cancer cytotoxicity of the lead compound 5a and attempted to establish similar biological activity of the derivatives of 5a to establish the best derivative for future medicinal chemistry efforts. Although the current series of 16 derivatives were not predicted to be blood–brain barrier penetrant, we explored the potential of combining a 6-amino-2-pyridone-3,5-dicarbonitrile with clinically relevant BBB-penetrant small molecules^18–22^ to establish potency for future medicinal chemistry efforts to optimise a BBB-penetrant drug.

## 2. Results and discussion

### 2.1 Chemistry

To synthesise 6-amino-2-pyridone-3,5-dicarbonitrile, we followed the same protocol used in our previously reported work.^9^*p*-Tolylidenemalononitrile 3a synthesised by reaction of *p*-tolualdehyde 1a and malononitrile 2. Some natural products like pyridine-2-carboxylic acid, betaine, guanidine hydrochloride and guanidine carbonate are used as a catalyst for the one-pot reaction. At room temperature, 1a and 2 were mixed with 10 mol% catalyst and stirred. When betaine or guanidine carbonate was used, the reaction mixture solidified within 5 minutes in a round bottom flask, however, the reaction was still incomplete, therefore, we added 1 ml of methanol to the reaction mixture and stirred it again for 10 minutes. Aldehyde was completely used but, guanidine carbonate produced 3a with water-soluble impurities. Whereas betaine produced 3a as a single product. Thus, betaine is a suitable catalyst for the first step.

**Fig. 1:**
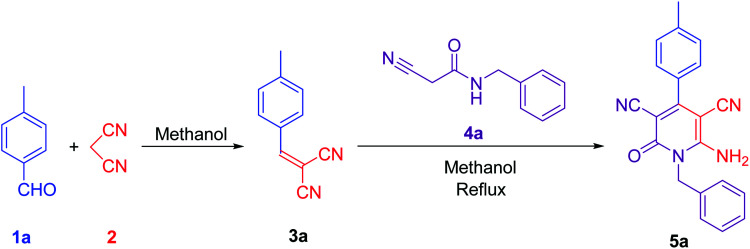
Synthesis of lead compound. Reaction condition: 2 mmol *p*-tolualdehyde, 2 mmol mole malononitrile, 2 mmol *N*-benzyl-2-cyanoacetamide in methanol solvent.

After completion of the first step with betaine catalyst, *N*-benzyl-2-cyanoacetamide 4a was added to the reaction mixture with 1 ml methanol. The reaction mixture was refluxed with pyridine-2-carboxylic acid, betaine, guanidine hydrochloride or guanidine carbonate catalyst. Betaine did not convert 3a to 5a completely in 5 minutes. However, guanidine carbonate efficiently catalysed this step and produced desired product 5a in 10 minutes. Thus, the one-pot two-step process is more suitable than the multi-component procedure for the synthesis of 6-amino-2-pyridone-3,5-dicarbonitrile derivatives using two catalysts: betaine for the first step and guanidine carbonate for the second step (Fig. 1 & Table 1).

**Table 1:** Optimisation of reaction

Entry	Catalyst (10 mol%)	Reaction time of first step	Conversion relative to aldehyde	Reaction time of second step	Conversion relative to 3a
1	Pyridine-2-carboxylic acid	30 min	Incomplete	1 h	Incomplete
2	Betaine	15 min	100%	1 h	Incomplete
3	Guanidine hydrochloride	30 min	Incomplete	1 h	Incomplete
4	Guanidine carbonate	10 min	100%	10 min	100%

**Fig. 2:**
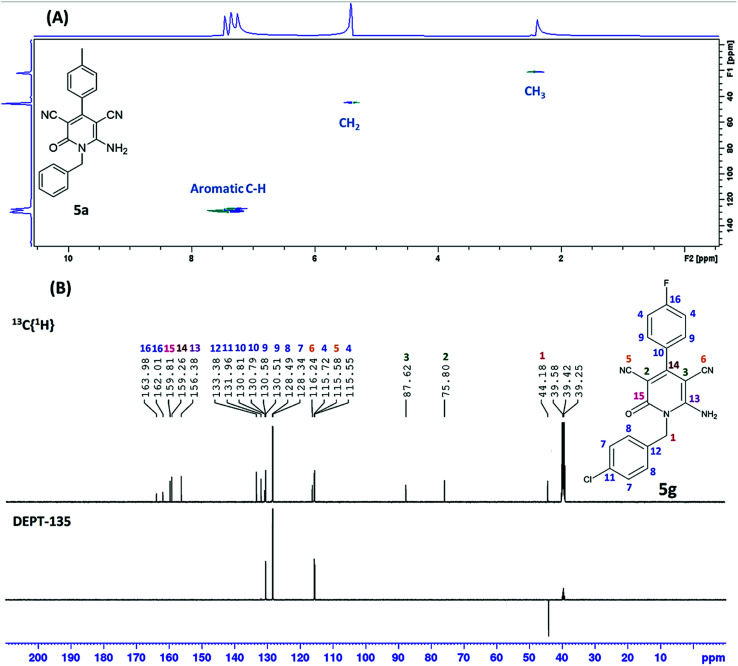
(A) HSQC spectrum of 5a, (B) DEPT-135 spectrum of 5g.

As shown in Table 2, sixteen 6-amino-2-pyridone-3,5-dicarbonitriles 5(a–p) were synthesised *via* a one-pot two-step reaction. 5(a–p) were characterised by ^1^H NMR, ^13^C{^1^H} NMR and HRMS analysis. Some of the products were also analysed by DEPT-135 or HSQC analysis. HSQC spectra confirmed the presence of benzyl group (C̲H_2_Ph), aromatic rings and methyl group in case of 5a (Fig. 2A). DEPT-135 spectra confirmed the presence of different types of carbons (Fig. 2B). Due to C–F coupling singles of C-16, C-4, C-9 and C-10 are observed as a doublet with coupling constant (*J*) as 247.77, 21.38, 8.80 and 2.52 Hz, respectively.

**Table 2:** Establishing biological activity of the derivatives of 5a^a^

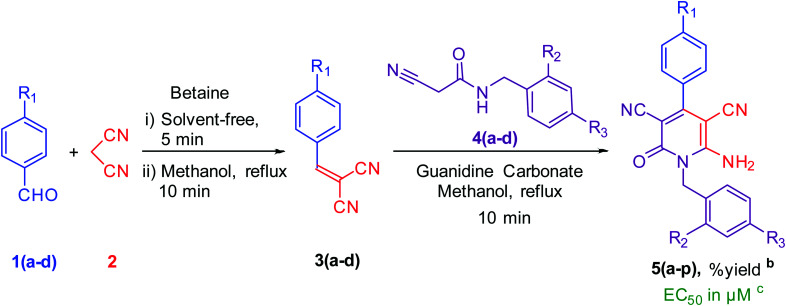
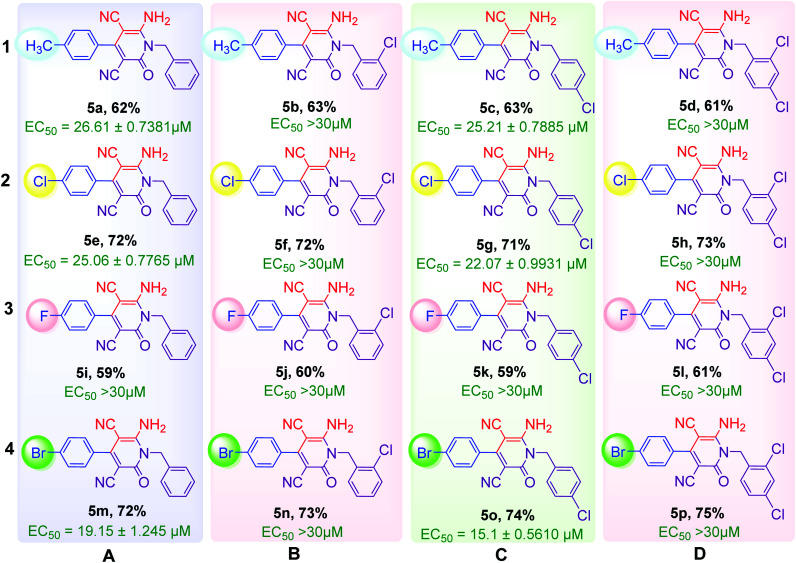

a Reaction condition: 2 mmol *p*-tolualdehyde, 2 mmol mole malononitrile, 2 mmol *N*-benzyl-2-cyanoacetamide, in methanol solvent.

b Isolated yield.

c EC_50_ against murine glioma GL261 and triplicates were performed for all assays.

We reported the synthesis of intermediate aryledinemalononitrile by betaine catalyst for the first time. However, other betaine base catalysts are reported for different heterocyclic reactions. We also tried the synthesis of aryledinemalononitrile by the solvent-free grinding method in the presence of betaine catalyst. Betaine successfully catalysed this room-temperature synthesis and produced intermediate 3 within 10 minutes. However, this solvent-free grinding method did not carry out the second step and hence expected product was not synthesised. All subsequent derivatives were synthesised using the one-pot two-step synthesis in the round bottom flask.

### 2.2 Biology

#### 2.2.1 Establishing a potential relationship between structure and biological activity of 5a derivatives

After characterisation, the *in vitro* cytotoxic activity of compound 5awas analysed by titrating the molecules at various concentrations against murine glioma GL261 cells. EC_50_ was determined by deriving a dose-response inhibition curve as 26.61 ± 0.7381 μM. In order to increase the GL261 anti-proliferative activity of lead compound 5a (EC_50_ = ∼27 μM), a reasonably optimised, focussed library of 16 derivatives was designed and synthesised (Table 1). Firstly, mono or di-chloro groups were introduced at *ortho* (R_2_) and/or *para* (R_3_) positions on benzyl group at position 1 of the 1,2-dihydropyridine-3,5-dicarbonitrile ring (5b–d). An introduction of chlorine at R3 modestly improved cytotoxicity of 5c. But the introduction of chlorine at R_3_ decreased the cytotoxicity of 5b and 5d. Next, the replacement of the methyl group with a chlorine group at para position (R_1_) of a phenyl ring of 5a increased the cytotoxicity of compounds. Furthermore, we also introduced other halogens like fluorine and bromine at R_1_ of the phenyl ring. Significant improvement was observed in the case of bromine substitution. But fluorine substitution decreased the cytotoxicity of compounds. In Table 2, cytotoxicity increases horizontally as B = D < A < C and vertically as 3 < 1 < 2 < 4. Intriguingly, introducing chlorine at R3 to the bromo derivative improved cytotoxicity by nearly 2-fold (5o) (EC_50_ = 15 μM). Together, we selected 5o to investigate its cytotoxic effects on diverse cancer cells.

#### 2.2.2 Cytotoxic activity against diverse cancer cell lines

**Fig. 3:**
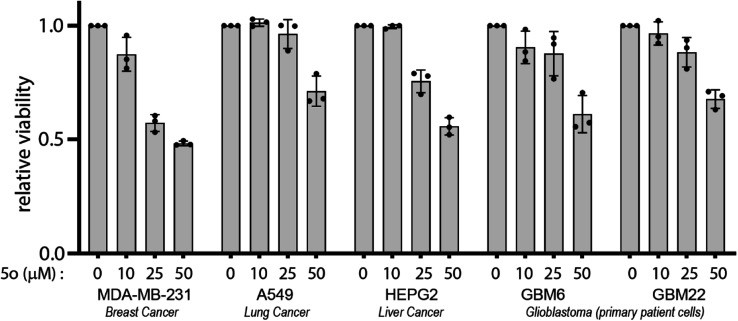
5o exhibits anti-cancer activity in diverse cancer cell lines. Indicated cell lines were treated with various doses of 5o for 72 hours and their viability was measured using CellTiter 96 AQueous Non-Radioactive Cell Proliferation Assay kit. Viability of DMSO-treated cells was used as control. Data are represented as fold viability of DMSO-treated control for each cell line with *n* = 3 biological replicates.

MDA-MB-231,^23^ A549 ^22^ and HEPG2 ^24^ cancer cell lines, as well as primary patient-derived glioblastoma cells GBM6 and GBM22, were used in order to assess the anti-cancer properties of the compounds *in vitro* using cell viability assays. MDA-MB-231 is a triple-negative breast cancer epithelial cell line, A549 is a non-small cell lung cancer cell line and HEPG2 is a hepatocellular carcinoma cell line (Fig. 3). GBM6 and GBM22 were established at the Mayo Clinic Brain Tumor PDX National Resource as discussed previously.^26^ The anti-cancer activity of 5o was measured in the aforementioned cell lines.

#### 2.2.3 Combination with clinically relevant brain-penetrant drugs

**Fig. 4:**
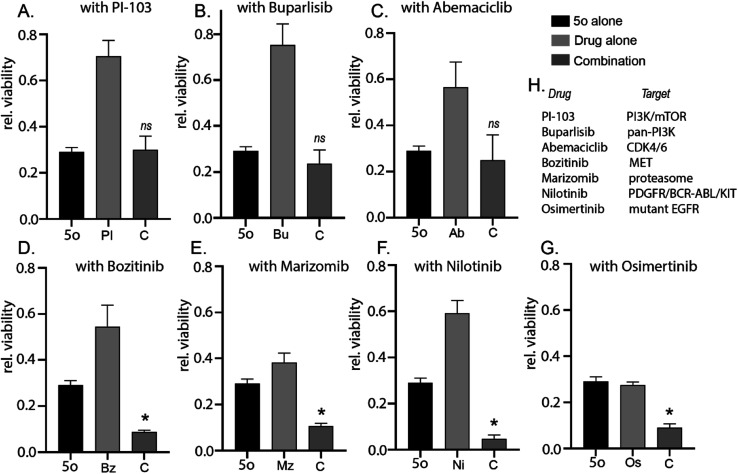
5o induces enhanced cytotoxicity in combination with specific clinically relevant brain-penetrant small molecule inhibitors. GL261 cells were treated with 5o alone (30 μM) or clinically relevant brain-penetrant drugs alone (A) PI-103 100 nm, (B) Buparlisib 500 nm, (C) Abemaciclib 1 μM, (D) Bozitinib 7 μM, (E) Marizomib 400 nM, (F) Nilotinib 5 μM, (G) Osimertinib 2 μM, or a combination of both for 72 hours and cell viability was analysed by CellTiter 96 AQueous Non-Radioactive Cell Proliferation Assay kit. Viability of DMSO-treated cells was used as control. Data are represented as fold viability of DMSO-treated control for each cell line (* indicates statistical significance compared to each single compound treatment; one-way ANOVA with Dunnett's multiple comparison; *ns*: not significant). Same dataset for 5o was used across all combination studies from (A–G). (H) Table lists the cellular targets of the respective clinically relevant brain-penetrant drugs.

To further explore the ability of 5o to induce cytotoxicity in cancer cells, we tested 5o in combination with PI-103, buparlisib, abemaciclib, bozitinib, marizomib, nilotinib, and osimertinib. In combination with PI3K and CDK4/6 inhibitory molecules, 5o did not exhibit any additive or synergistic cytotoxicity (Fig. 4A–C). Interestingly, 5o induced potent cytotoxicity in combination with bozitinib, marizomib, nilotinib, and osimertinib (Fig. 4D–G). This suggests that future brain-penetrant derivatives of 5o could be used in combination with receptor-tyrosine kinase or proteasome inhibitors to treat brain tumours.

## 3. Conclusion

A series of novel 6-amino-2-pyridone-3,5-dicarbonitrile derivatives were synthesised using a one-pot two-step scheme using natural product catalysts. Out of the library we identified 5o with the most potent anti-cancer activity in a diverse set of cancer cell lines including primary patient-derived cells. Furthermore, in combination with brain-penetrant small molecule inhibitors targeting receptor tyrosine kinases and proteasome, 5o induced potent cytotoxicity which attests for the further medicinal chemistry and development of the 6-amino-2-pyridone-3,5-dicarbonitrile backbone. Therefore, this study establishes a simple yet novel synthetic scheme which will allow for the development of future clinically relevant anti-cancer molecules which can be used either as a monotherapeutic option or used in combination with specific molecules targeting diverse cancer signalling pathways.

## 4. Experimental procedure

### 4.1 Chemistry

#### 4.1.1 General

All chemicals were purchased from commercially available sources and used without further purification. *N*-Benzyl-2-cyanoacetamide and its chloro derivatives were synthesised following a previously reported procedure.^25^ Melting points were determined by the open capillary tube method and are uncorrected. ^1^H NMR and ^13^C{^1^H} NMR and HSQC spectral analysis were recorded on BRUKER AVANCE II 600 NMR Spectrometer using DMSO-d_6_ as the solvents and TMS as the internal standard. Splitting patterns of an apparent multiplet associated with an averaged coupling constant were designated as follows: s = singlet, d = doublet, t = triplet, dd = double doublet, ABq = AB quartet and m = multiplet. The chemical shifts are expressed in parts per million and coupling constants (*J*) are provided in Hertz. In ^1^H-NMR spectrum which run in DMSO-d_6_ solvent, chemical shifts of trace solvent impurities were also observed. For example, residual peak of water was observed at *δ* ∼ 3.33 ppm. Residual peaks of ethanol were observed at *δ* ∼ 1.06, ∼3.44 and ∼4.35 ppm. Residual peaks of methanol were observed at *δ* ∼3.17 and ∼4.10 ppm.

#### 4.1.2 General procedure for the synthesis of 5(a–p)

A mixture of 2 mmol aldehyde, 2 mmol malononitrile and 0.2 mmol was added to the round bottom flask and stirred with a tiny magnetic needle for 5 minutes in solvent-free condition. 1 ml methanol was added to the reaction mixture and refluxed the reaction mixture for 10 minutes. 2 mmol *N*-substituted 2-cyanoacetamides and 0.2 mmol guanidinium carbonate were added with 2 ml methanol and the reaction mixture was refluxed for 10 minutes. The reaction mixture was cooled in ice. 10 ml ethyl acetate was added to the reaction mixture and pour into a separating funnel containing 10 ml water with 1 g table salt. The separating funnel was shaken for 5–7 minutes, and ethyl acetate layer was separated. Ethyl acetate was evaporated under vacuum distillation. Recrystallised the product in methanol or ethanol solvent.

##### 4.1.2.1 6-Amino-1-benzyl-2-oxo-4-*p*-tolyl-1,2-dihydropyridine-3,5-dicarbonitrile (5a)

White solid (0.423 g, 62%), mp: 292–294 °C; ^1^H NMR (600 MHz, DMSO-d_6_) *δ*: 2.42 (s, 3H, CH_3_), 5.39 (s, 2H, CH_2_), 7.28 and 7.49 (ABq, *J* = 8.1 Hz, 2H, Ar–H), 7.32 (t, *J* = 7.2 Hz, 1H, Ar–H), 7.39 (d, *J* = 5.4 Hz, 3H, Ar–H), 8.46 (s, 2H, NH_2_); ^13^C{^1^H} NMR (151 MHz, DMSO-d_6_) *δ*: 21.5, 45.2, 76.1, 88.0, 116.4, 117.0, 127.0, 127.9, 128.5, 129.0, 129.6, 132.2, 135.0, 140.7, 157.1, 160.0, 161.35; MS (ESI-TOF) *m*/*z* calcd For C_21_H_16_N_4_O (M + H)^+^: 341.1402, found: 341.1420.

##### 4.1.2.2. 6-Amino-1-(2-chlorobenzyl)-2-oxo-4-*p*-tolyl-1,2-dihydropyridine-3,5-dicarbonitrile (5b)

White solid (0.473 g, 63%), mp: 292–294 °C; ^1^H NMR (600 MHz, DMSO-d_6_) *δ*: 2.40 (s, 3H, CH_3_), 5.28 (s, 2H, CH_2_), 6.91 (d, *J* = 6 Hz, 1H, Ar–H), 7.31–7.39 (m, 4H, Ar–H), 7.47–7.54 (m, 3H, Ar–H), 8.53 (s, 2H, NH_2_); ^13^C{^1^H} NMR (151 MHz, DMSO-d_6_) *δ*: 21.5, 44.8, 76.3, 87.8, 116.5, 117.0, 125.8, 127.9, 128.5, 129.3, 129.6, 130.0, 132.2, 132.4, 132.7, 140.7, 157.4, 159.8, 161.6; MS (ESI-TOF) *m*/*z* calcd for C_21_H_15_ClN_4_O (M + H)^+^: 375.1013, found: 375.1028.

##### 4.1.2.3 6-Amino-1-(4-chlorobenzyl)-2-oxo-4-*p*-tolyl-1,2-dihydropyridine-3,5-dicarbonitrile (5c)

White solid (0.472 g, 63%), mp: 250–252 °C; ^1^H NMR (600 MHz, DMSO-d_6_) *δ*: 2.40 (s, 3H, CH_3_), 5.35 (s, 2H, CH_2_), 7.31 (d, *J* = 8.4 Hz, 2H, Ar–H), 7.38 (d, *J* = 7.8 Hz, 2H, Ar–H), 7.45 (dd, *J* = 8.4, 13.8 Hz, 2H, Ar–H), 8.46 (s, 2H, NH_2_); ^13^C{^1^H} NMR (151 MHz, DMSO-d_6_) *δ*: 21.5, 44.8, 76.2, 88.0, 116.3, 117.0, 128.5, 128.9, 129.1, 129.6, 132.10, 132.6, 134.0, 140.7, 157.0, 160.0, 161.4; MS (ESI-TOF) *m*/*z* calcd for C_21_H_15_ClN_4_O (M + H)^+^: 375.1013, found: 375.0982.

##### 4.1.2.4 6-Amino-1-(2,4-dichlorobenzyl)-2-oxo-4-*p*-tolyl-1,2-dihydropyridine-3,5-dicarbonitrile (5d)

White solid (0.499 g, 61%), mp: 314–316 °C; ^1^H NMR (600 MHz, DMSO-d_6_) *δ*: 2.42 (s, 3H, CH_3_), 5.23 (s, 2H, CH_2_), 6.98 (d, *J* = 7.8 Hz, 1H, Ar–H), 7.39–7.46 (m, 5H, Ar–H), 7.73 (s, 1H, Ar–H), 8.58 (s, 2H, NH_2_); ^13^C{^1^H} NMR (151 MHz, DMSO-d_6_) *δ*: 21.5, 44.6, 76.4, 87.8, 116.4, 116.9, 127.3, 127.9, 128.5, 129.4, 129.6, 131.7, 132.2, 133.0, 133.6, 140.7, 157.4, 159.7, 161.6; MS (ESI-TOF) *m*/*z* calcd for C_21_H_14_Cl_2_N_4_O (M + H)^+^: 409.0623, found: 409.0844.

##### 4.1.2.5 6-Amino-1-benzyl-4-(4-chlorophenyl)-2-oxo-1,2-dihydropyridine-3,5-dicarbonitrile (5e)

White solid (0.519 g, 72%), mp: 272–274 °C; ^1^H NMR (500 MHz, DMSO-d_6_) *δ*: 5.35 (s, 2H, CH_2_), 7.25 (d, *J* = 7.5 Hz, 2H, Ar–H), 7.31 (t, *J* = 7.25, 1H, Ar–H), 7.38 (t, *J* = 7.25, 2H, Ar–H), 7.60 (dd, *J* = 2.0, 6.5 Hz, 2H, Ar–H), 7.66 (dd, *J* = 2.0, 6.5 Hz, 2H, Ar–H), 8.49 (s, 2H, NH_2_); ^13^C{^1^H} NMR (125 MHz, DMSO-d_6_) *δ*: 44.6, 75.6, 87.4, 115.5, 116.2, 126.4, 127.3, 128.4, 128.7, 129.9, 133.3, 134.3, 135.1, 156.4, 159.2, 159.5; MS (ESI-TOF) *m*/*z* calcd for C_20_H_13_ClN_4_O (M + H)^+^: 361.0856, found: 361.0871.

##### 4.1.2.6 6-Amino-1-(2-chlorobenzyl)-4-(4-chlorophenyl)-2-oxo-1,2-dihydropyridine-3,5-dicarbonitrile (5f)

White solid (0.569 g, 72%), mp: 284–286 °C; ^1^H NMR (600 MHz, DMSO-d_6_) *δ*: 5.28 (s, 2H, CH_2_), 6.93 (d, *J* = 7.8 Hz, 1H, Ar–H), 7.39 (dt, *J* = 7.2, 22.8 Hz, 2H, Ar–H), 7.55–7.56 (m, 1H, Ar–H), 7.62 (d, *J* = 8.4 Hz, 1H, Ar–H), 7.68 (d, *J* = 9.0 Hz, 1H, Ar–H), 8.61 (s, 2H, NH_2_); ^13^C{^1^H} NMR (151 MHz, DMSO-d_6_) *δ*: 44.8, 76.3, 87.9, 116.2, 116.7, 125.8, 127.8, 129.3, 130.0, 130.5, 132.2, 132.7, 134.0, 135.7, 157.8, 159.6, 160.4; MS (ESI-TOF) *m*/*z* calcd for C_20_H_12_Cl_2_N_4_O (M + H)^+^: 395.0466, found: 395.0511.

##### 4.1.2.7 6-Amino-1-(4-chlorobenzyl)-4-(4-chlorophenyl)-2-oxo-1,2-dihydropyridine-3,5-dicarbonitrile (5g)

White solid (0.561 g, 71%), mp: 244–246 °C; ^1^H NMR (600 MHz, DMSO-d_6_) *δ*: 5.32 (s, 2H, CH_2_), 7.29 (d, *J* = 8.4 Hz, 2H, Ar–H), 7.44 (d, *J* = 8.4 Hz, 2H, Ar–H), 7.59 (d, *J* = 8.4 Hz, 2H, Ar–H), 7.66 (d, *J* = 9.0 Hz, 2H, Ar–H), 8.51 (s, 2H, NH_2_); ^13^C{^1^H} NMR (151 MHz, DMSO-d_6_) *δ*: 44.8, 76.2, 88.1, 116.1, 116.7, 128.9, 129.1, 129.3, 130.5, 132.6, 133.9, 133.9, 135.7, 157.0, 159.8, 160.2; MS (ESI-TOF) *m*/*z* calcd for C_20_H_12_Cl_2_N_4_O (M + H)^+^: 395.0466, found: 395.0476.

##### 4.1.2.8 6-Amino-4-(4-chlorophenyl)-1-(2,4-dichlorobenzyl)-2-oxo-1,2-dihydropyridine-3,5-dicarbonitrile (5h)

White solid (0.627 g, 73%), mp: 292–294 °C (d); ^1^H NMR (600 MHz, DMSO-d_6_) *δ*: 5.23 (s, 2H, CH_2_), 6.99 (d, *J* = 8.4 Hz, 1H, Ar–H), 7.39 (dd, *J* = 1.5, 8.1 Hz, 1H, Ar–H), 7.61 (d, *J* = 8.4 Hz, 2H, Ar–H), 7.68 (d, *J* = 8.4 Hz, 2H, Ar–H), 7.73 (d, *J* = 1.2 Hz, 1H, Ar–H), 8.62 (s, 2H, NH_2_); ^13^C{^1^H} NMR (151 MHz, DMSO-d_6_) *δ*: 44.7, 76.4, 87.9, 116.2, 116.7, 127.3, 127.9, 129.3, 129.5, 130.5, 131.6, 133.0, 133.7, 133.9, 135.7, 157.4, 159.6, 160.4; MS (ESI-TOF) *m*/*z* calcd for C_20_H_11_Cl_3_N_4_O (M + H)^+^: 429.0077, found: 429.0107.

##### 4.1.2.9 6-Amino-1-benzyl-4-(4-fluorophenyl)-2-oxo-1,2-dihydropyridine-3,5-dicarbonitrile (5i)

White solid (0.406 g, 59%), mp: 228–230 °C; ^1^H NMR (500 MHz, DMSO-d_6_) *δ*: 5.35 (s, 2H, CH_2_), 7.25 (d, *J* = 9 Hz, 2H, Ar–H), 7.30–7.36 (m, 1H, Ar–H), 7.37–7.44 (m, 4H, Ar–H), 7.63–7.66 (m, 2H, Ar–H), 8.47 (s, 2H, NH_2_); ^13^C{^1^H} NMR (125 MHz, DMSO-d_6_) *δ*: 44.6, 75.7, 87.6, 115.5, 115.6, 115.7, 116.3, 126.4, 127.3, 128.4, 130.5, 130.6, 130.8, 130.9, 134.3, 156.4, 159.3, 159.8, 162.0, 164.0; MS (ESI-TOF) *m*/*z* calcd for C_20_H_13_FN_4_O (M + H)^+^: 345.1152, found: 345.1145.

##### 4.1.2.10 6-Amino-1-(2-chlorobenzyl)-4-(4-fluorophenyl)-2-oxo-1,2-dihydropyridine-3,5-dicarbonitrile (5j)

White solid (0.454 g, 60%), mp: 298–300 °C; ^1^H NMR (600 MHz, DMSO-d_6_) *δ*: 5.27 (s, 2H, CH_2_), 6.92 (d, *J* = 7.8 Hz, 1H, Ar–H), 7.32–7.38 (m, 2H, Ar–H), 7.45 (t, *J* = 8.7 Hz, 2H, Ar–H), 7.55 (d, *J* = 7.2 Hz, 1H, Ar–H), 7.65 (dd, *J* = 5.7, 8.1 Hz, 2H, Ar–H), 8.58 (s, 2H, NH_2_); ^13^C{^1^H} NMR (151 MHz, DMSO-d_6_) *δ*: 44.7, 76.5, 88.0, 116.2, 116.3, 116.3, 116.8, 125.8, 127.9, 129.3, 130.0, 131.1, 131.2, 131.5, 132.2, 132.6, 157.4, 159.6, 160.6, 162.8, 164.4; MS (ESI-TOF) *m*/*z* calcd for C_20_H_12_ClFN_4_O (M + H)^+^: 379.0762, found: 379.0756.

##### 4.1.2.11 6-Amino-1-(4-chlorobenzyl)-4-(4-fluorophenyl)-2-oxo-1,2-dihydropyridine-3,5-dicarbonitrile (5k)

White solid (0.447 g, 59%), mp: 130–132 °C; ^1^H NMR (500 MHz, DMSO-d_6_) *δ*: 5.32 (s, 2H, CH_2_), 7.28 (d, *J* = 10.2 Hz, 2H, Ar–H), 7.41–7.45 (m, 4H, Ar–H), 7.61–7.64 (m, 2H, Ar–H), 8.48 (s, 2H, NH_2_); ^13^C{^1^H} NMR (125 MHz, DMSO-d_6_) *δ*: 44.2, 75.8, 87.6, 115.6, 115.6, 115.7, 116.2, 128.3, 128.5, 130.5, 130.6, 130.8, 130.8, 132.0, 133.4, 156.4, 159.3, 159.8, 162.0, 164.0; MS (ESI-TOF) *m*/*z* calcd for C_20_H_12_ClFN_4_O (M + H)^+^: 379.0762, found: 379.0768.

##### 4.1.2.12 6-Amino-1-(2,4-dichlorobenzyl)-4-(4-fluorophenyl)-2-oxo-1,2-dihydropyridine-3,5-dicarbonitrile (5l)

White solid (0.504 g, 61%), mp: 278–280 °C; ^1^H NMR (600 MHz, DMSO-d_6_) *δ*: 5.22 (s, 2H, CH_2_), 6.98 (d, *J* = 8.4 Hz, 1H, Ar–H), 7.39 (d, *J* = 7.8 Hz, 1H, Ar–H), 7.45 (t, *J* = 8.4 Hz, 2H, Ar–H), 7.63–7.66 (m, 2H, Ar–H), 7.73 (s, 1H, Ar–H), 8.59 (s, 2H, NH_2_); ^13^C{^1^H} NMR (151 MHz, DMSO-d_6_) *δ*: 44.6, 76.5, 88.0, 116.2, 116.2, 116.3, 116.8, 127.3, 127.9, 129.5, 131.1, 131.1, 131.4, 131.6, 133.0, 133.6, 157.8, 159.6, 160.6, 162.8, 164.4; MS (ESI-TOF) *m*/*z* calcd for C_20_H_11_Cl_2_FN_4_O (M + H)^+^: 413.0372, found: 413.0603.

##### 4.1.2.13 6-Amino-1-benzyl-4-(4-bromophenyl)-2-oxo-1,2-dihydropyridine-3,5-dicarbonitrile (5m)

White solid (0.584 g, 72%), mp: 280–282 °C; ^1^H NMR (500 MHz, DMSO-d_6_) *δ*: 5.34 (s, 2H, CH_2_), 7.25 (d, *J* = 7.5 Hz, 2H, Ar–H), 7.31 (t, *J* = 7.5 Hz, 1H, Ar–H), 7.37–7.40 (m, 3H, Ar–H), 7.52–7.54 (m, 2H, Ar–H), 7.79–7.81 (m, 2H, Ar–H), 8.49 (s, 2H, NH_2_); ^13^C{^1^H} NMR (125 MHz, DMSO-d_6_) *δ*: 44.6, 75.5, 87.4, 115.5, 116.2, 123.8, 126.4, 127.3, 128.4, 130.1, 131.6, 133.7, 134.2, 156.4, 159.2, 159.6; MS (ESI-TOF) *m*/*z* calcd for C_20_H_13_BrN_4_O (M + H)^+^: 405.0351, found: 405.0569.

##### 4.1.2.14 6-Amino-4-(4-bromophenyl)-1-(2-chlorobenzyl)-2-oxo-1,2-dihydropyridine-3,5-dicarbonitrile (5n)

White solid (0.642 g 73%), mp: 290–292 °C; ^1^H NMR (600 MHz, DMSO-d_6_) *δ*: 5.27 (s, 2H, CH_2_), 6.93 (d, *J* = 7.2 Hz, 1H, Ar–H), 7.35 (dt, *J* = 7.5, 23.4 Hz, 2H, Ar–H), 7.55 (dd, *J* = 4.5, 6.9 Hz, 3H, Ar–H), 7.82 (d, *J* = 7.8 Hz, 2H, Ar–H), 8.61 (s, 2H, NH_2_); ^13^C{^1^H} NMR (151 MHz, DMSO-d_6_) *δ*: 44.8, 76.3, 87.8, 116.2, 116.7, 124.4, 125.8, 127.9, 129.3, 130.0, 130.5, 130.7, 132.2, 132.6, 134.4, 157.4, 159.6, 160.4; MS (ESI-TOF) *m*/*z* calcd for C_20_H_12_BrClN_4_O (M + H)^+^: 438.9961, found: 439.0189.

##### 4.1.2.15 6-Amino-4-(4-bromophenyl)-1-(4-chlorobenzyl)-2-oxo-1,2-dihydropyridine-3,5-dicarbonitrile (5o)

White solid (0.650 g, 74%), mp: 260–262 °C; ^1^H NMR (600 MHz, DMSO-d_6_) *δ*: 5.32 (s, 2H, CH_2_), 7.28 (d, *J* = 10.2 Hz, 2H, Ar–H), 7.41–7.45 (m, 4H, Ar–H), 7.61–7.64 (m, 2H, Ar–H), 8.48 (s, 2H, NH_2_); ^13^C{^1^H} NMR (151 MHz, DMSO-d_6_) *δ*: 44.8, 76.2, 88.0, 116.1, 116.7, 124.5, 128.9, 129.1, 130.7, 132.2, 132.6, 133.9, 134.2, 157.0, 159.8, 160.2; MS (ESI-TOF) *m*/*z* calcd for C_20_H_12_BrClN_4_O (M + H)^+^: 438.9961, found: 439.0183.

##### 4.1.2.16 6-Amino-4-(4-bromophenyl)-1-(2,4-dichlorobenzyl)-2-oxo-1,2-dihydropyridine-3,5-dicarbonitrile (5p)

White solid (0.714 g, 75%), mp: 284–286 °C; 1H NMR (600 MHz, DMSO-d_6_) *δ*: 5.22 (s, 2H, CH_2_), 6.98 (d, *J* = 8.4 Hz, 1H, Ar–H), 7.39 (dd, *J* = 1.8, 8.4 Hz, 1H, Ar–H), 7.53 (d, *J* = 8.4 Hz, 2H, Ar–H), 7.73 (d, *J* = 1.8 Hz, 1H, Ar–H), 7.82 (d, *J* = 8.4 Hz, 2H, Ar–H), 8.61 (s, 2H, NH_2_); 13C{1H} NMR (151 MHz, DMSO-d_6_) *δ*: 44.7, 76.3, 87.8, 116.2, 116.7, 124.5, 127.3, 127.9, 129.5, 130.7, 131.6, 132.2, 133.0, 133.6, 134.3, 157.4, 159.6, 160.5; MS (ESI-TOF) *m*/*z* calcd for C_20_H_11_BrCl_2_N_4_O (M + H)^+^: 472.9572, found: 472.9563.

### 4.2 Cell based assays

#### 4.2.1 Materials

Drugs PI-103 (#A2067-APE), Bupalisib (#ORB669009-BOR), Abemaciclib (#S5716-SEL), Bozitinib (#S6762-SEL), Nilotinib (#S1033-SEL) and Osimertinib (#S7297-SEL) were from Stratech, UK. Marizomib (#SML1916-100UG) was from Sigma Millipore. The compounds were dissolved in DMSO to a working stock of 10 mM. MDA-MB-231, A549, and HEPG2 cells were from ATCC. GL261 was a kind gift from Dr Kun-Liang Guan (UC San Diego, USA). GBM6 and GBM22 were acquired from the Brain Tumour PDX National Resource, Mayo Clinic, USA. Insulin and epidermal growth factor were purchased from Sigma Millipore.

#### 4.2.2 Cell culture and cytotoxicity assays

Cells were grown in a humidified incubator with 5% CO_2_ at 37 °C. MDA-MB-231, A549, HEPG2 and GL261 cell lines were cultured in Dulbecco's modified Eagle's medium (DMEM, Gibco) supplemented with 10% FBS and 1% penicillin and streptomycin. Primary patient glioblastoma cell lines (GBM6 and GBM22) were cultured in DMEM supplemented with 10% FBS, 1% penicillin and streptomycin, 10 μg ml^−1^ insulin, and 20 ng ml^−1^ hEGF.

5000–8000 cells were plated per well in 96-well plate. 24 hours post-plating, the appropriate drug was added to the cells in triplicate at varying concentrations with a DMSO control. 72 hours after treatment, cell viability was then determined using CellTiter 96® AQueous Non-Radioactive Cell proliferation assay, adhering to manufacturer instructions. Absorbance was measured using a Tecan multi-well plate reader and data was represented relative to DMSO treated control.

#### 4.2.3 Statistical analysis

All analysis was conducted using Graphpad Prism statistical package and presented as mean ± SD unless otherwise stated. Figure legends contain details of the statistical tests and multiple comparisons conducted throughout. Experiments were repeated 2–3 times with multiple technical replicates in order for the appropriate statistical tests to be conducted.

## Conflicts of interest

The authors declare no conflicts of interest.

## Supplementary Material

RA-012-D2RA03579K-s001
