# Supplementary material for: One-pot two-step catalytic synthesis of 6-amino-2-pyridone-3,5-dicarbonitriles enabling anti-cancer bioactivity
Source: RSC Adv. 2022 Aug 24;12(37):23889–97. doi: 10.1039/d2ra03579k (PMC9400646; doi:10.1039/d2ra03579k)
Supplement: RA-012-D2RA03579K-s001 [file RA-012-D2RA03579K-s001.pdf]

Electronic Supplementary Material (ESI) for RSC Advances.
This journal is © The Royal Society of Chemistry 2022

Supporting Information

One-Pot Two-Step Catalytic Synthesis of Rationally Designed 6-amino-2-pyridone-3,5-dicarbonitriles Enabling Anti-Cancer Bioactivity

Lynden G. Nicely[1,4], Ruturajsinh M. Vala[2,4], Dipti B. Upadhyay[2], Joaquina Nogales[1], Celestine Chi[3], Sourav Banerjee[1,*], Hitendra M. Patel[2,*]

[1]Department of Cellular and Systems Medicine, School of Medicine, University of Dundee, Dundee DD1 9SY, UK.

[2]Department of Chemistry, Sardar Patel University, Vallabh Vidyanagar 388120, Gujarat, India.

[3] Department of Medical Biochemistry and Microbiology, Uppsala University, SE-75123 Uppsala, Sweden

[4]contributed equally

*Corresponding authors, SB: s.y.banerjee@dundee.ac.uk; HMP: hm_patel@spuvvn.edu

Table of Contents

Sr No.	Contents	Page No.
1	Copies of 1D and 2D NMR spectra of **5(a-p)**	S2-S24
2	Copies of HRMS of **5(a-p)**	S25-S30

1. Copies of 1D and 2D NMR spectra of **5(a-p)**

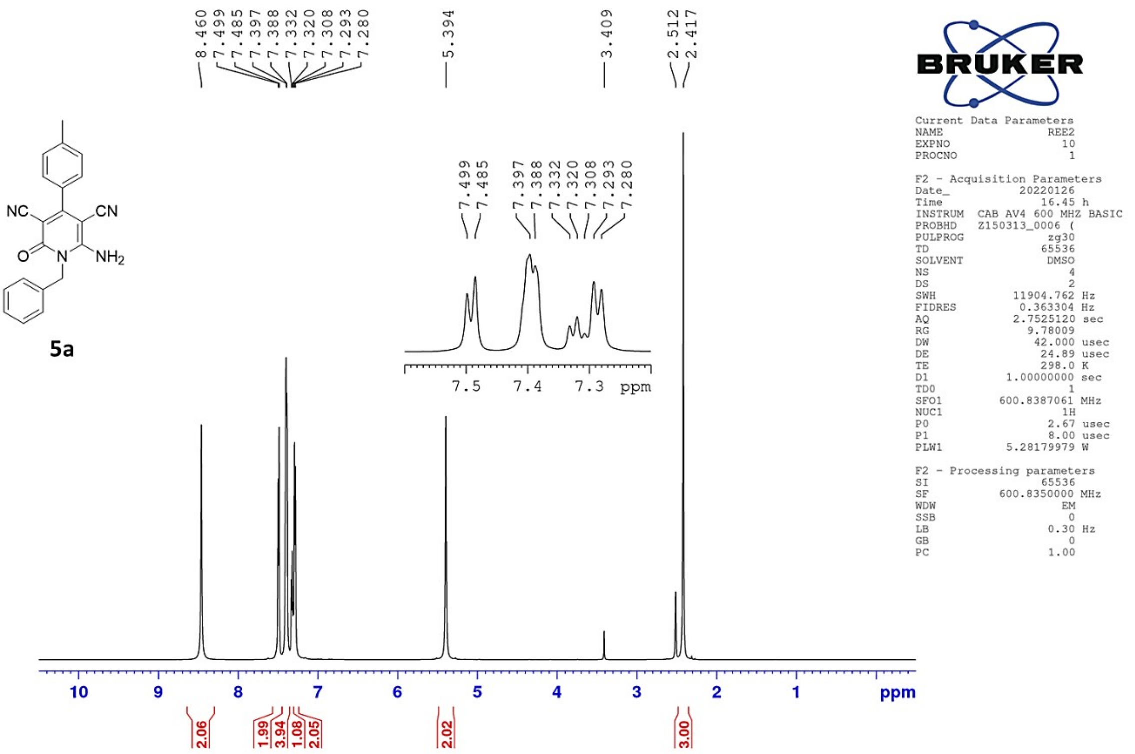

Figure S1. ^{1}H NMR spectrum of compound **5a** at 600 MHz in DMSO-d_6

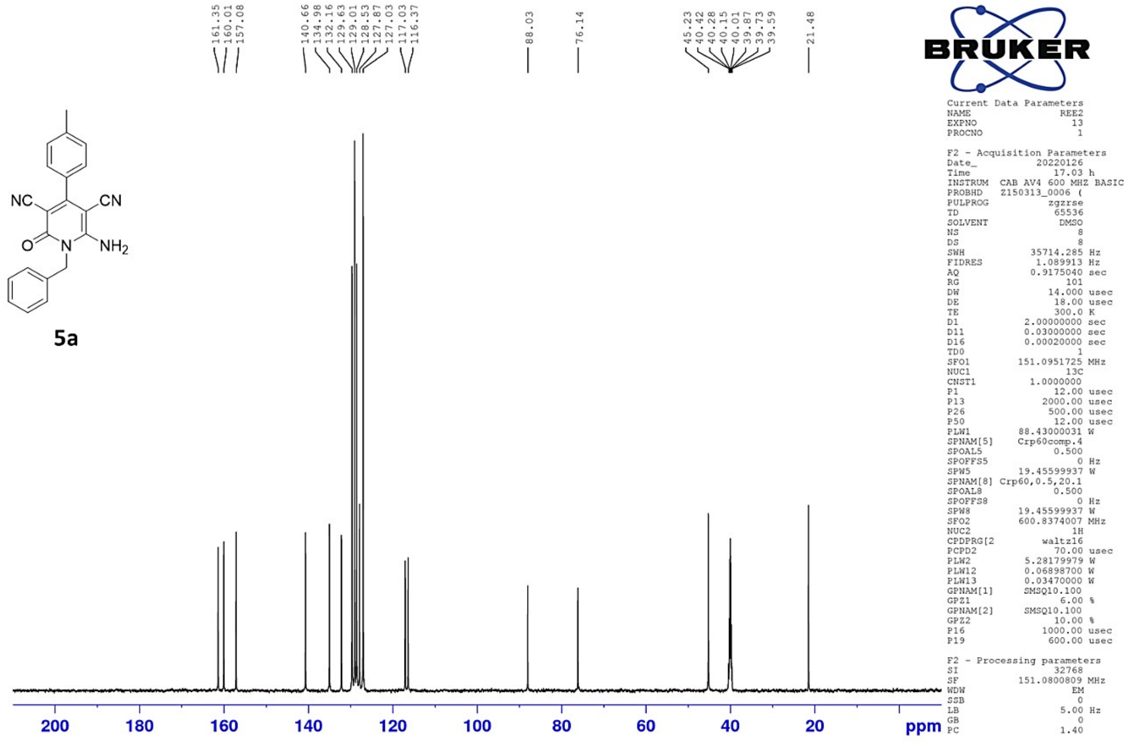

Figure S2. $^{13}C\{^{1}H\}$ NMR spectrum of compound **5a** at 151 MHz in DMSO-d_6

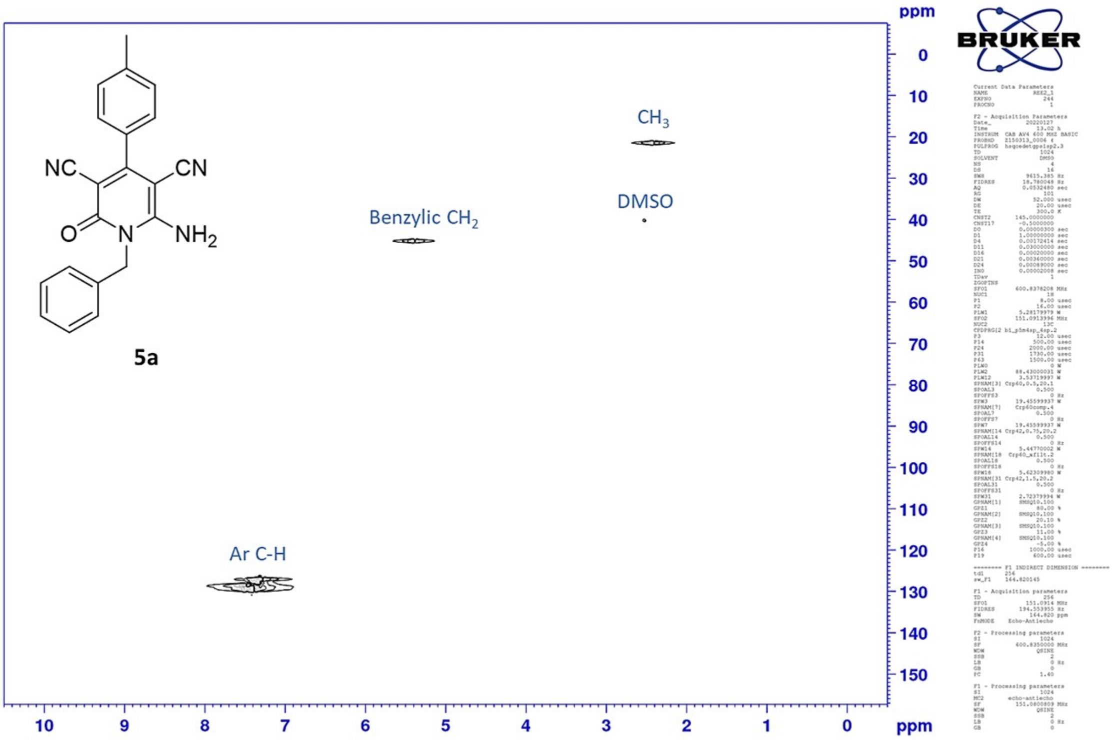

Figure S3. HSQC spectrum of compound **5a** in DMSO-d$_6$

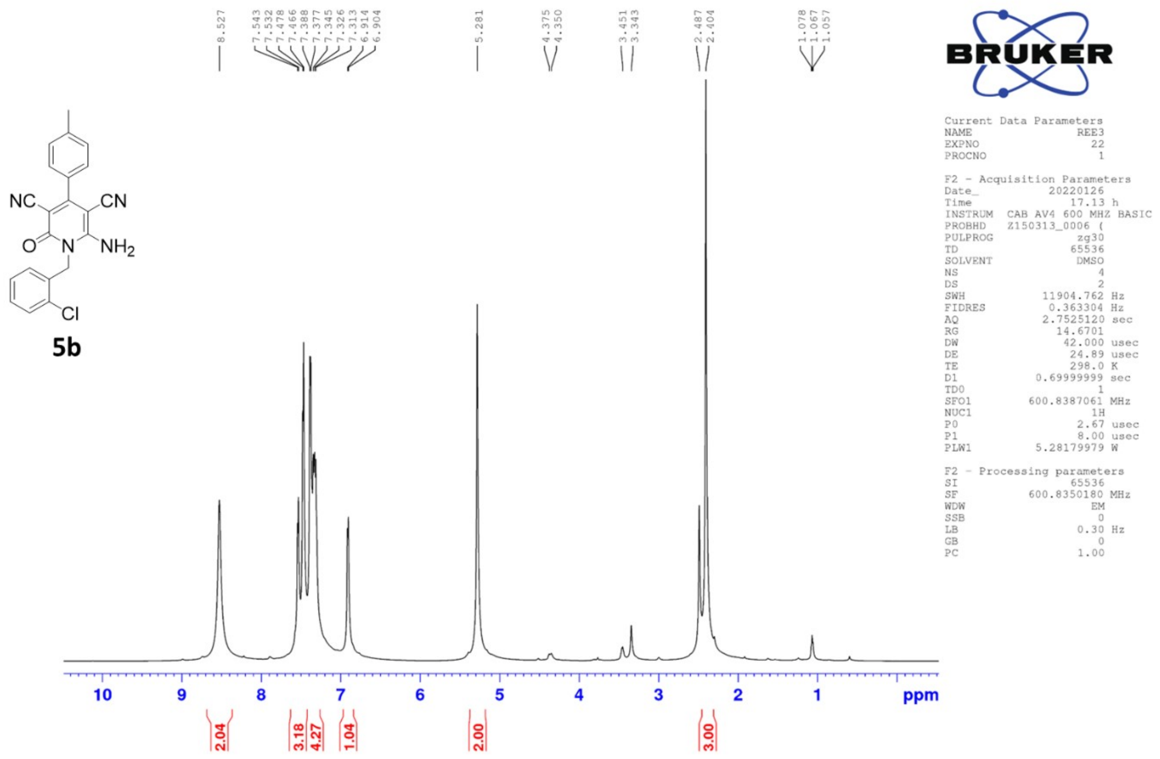

Figure S4. ^{1}H NMR spectrum of compound **5b** at 600 MHz in DMSO-d$_6$

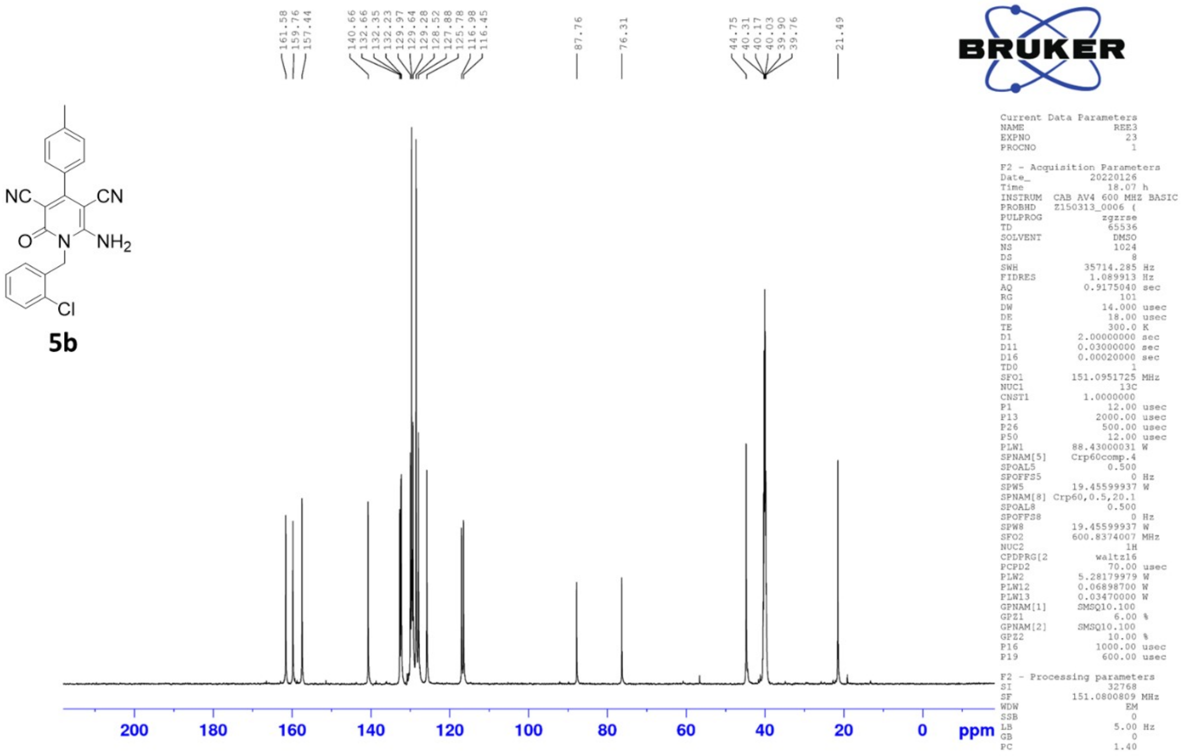

Figure S5. $^{13}C\{^{1}H\}$ NMR spectrum of compound **5b** at 151 MHz in DMSO-d_6

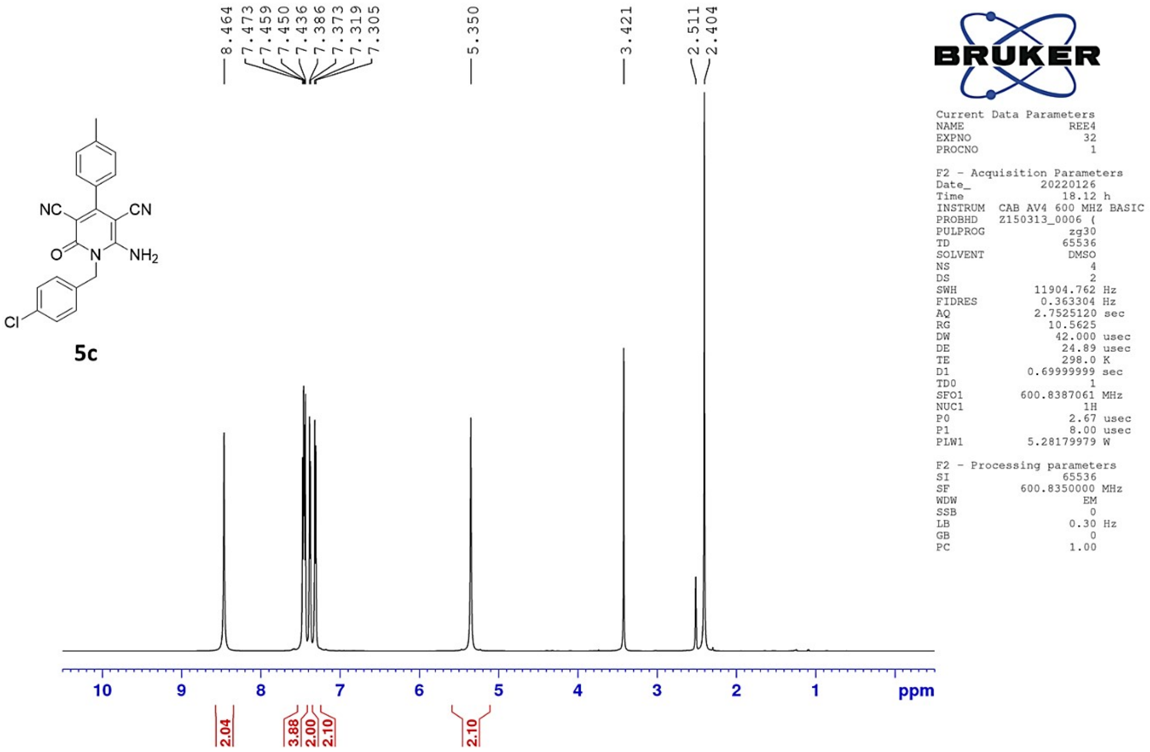

Figure S6. ^{1}H NMR spectrum of compound **5c** at 600 MHz in DMSO-d_6

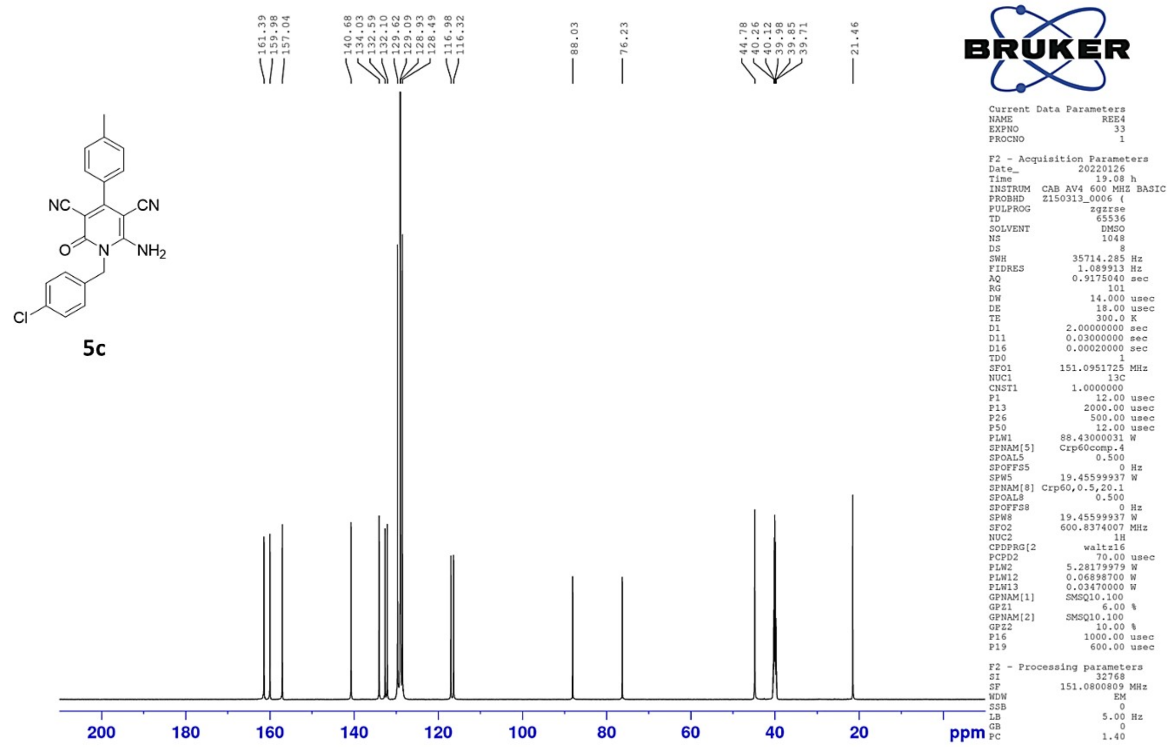

Figure S7. $^{13}C\{^{1}H\}$ NMR spectrum of compound **5c** at 151 MHz in DMSO-d_6

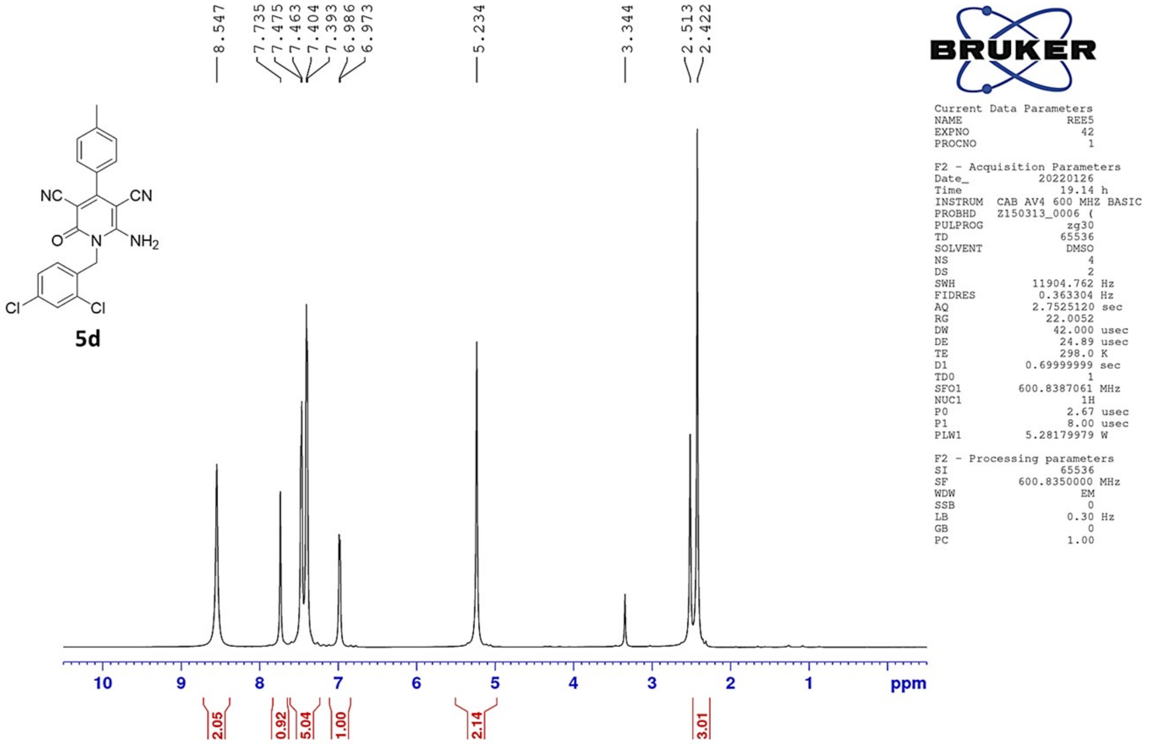

Figure S8. ^{1}H NMR spectrum of compound **5d** at 600 MHz in DMSO-d_6

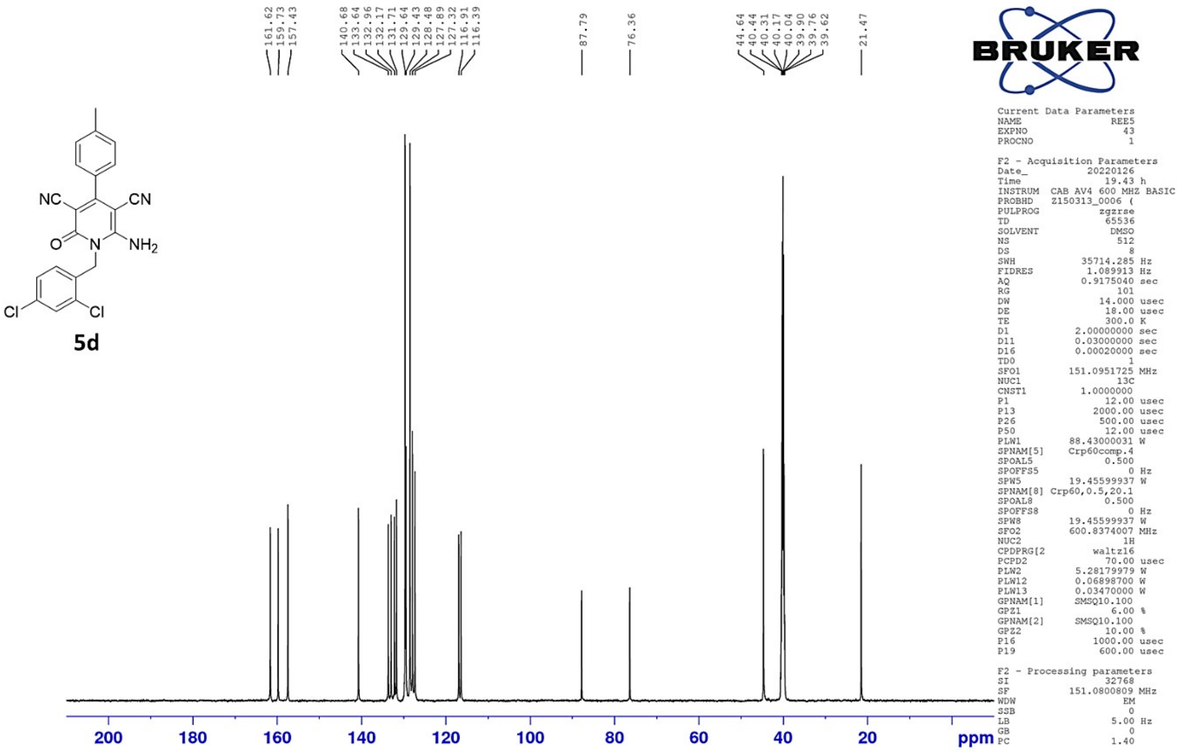

Figure S9. $^{13}C\{^{1}H\}$ NMR spectrum of compound **5d** at 151 MHz in DMSO-d_6

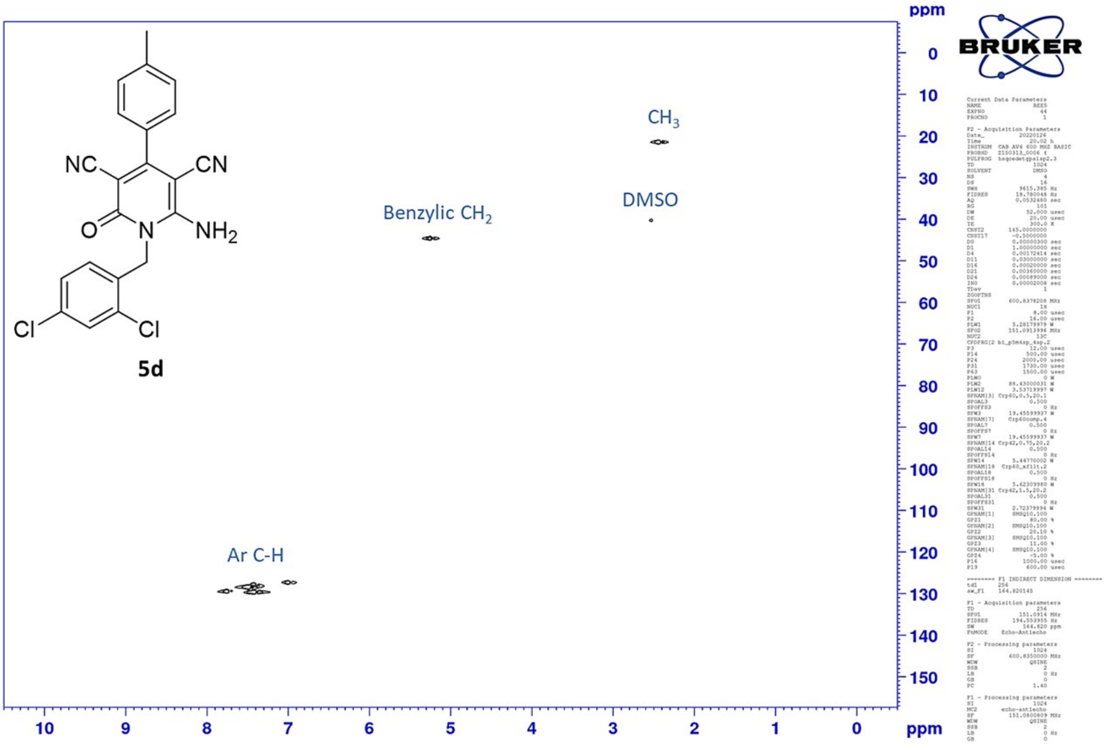

Figure S10. HSQC spectrum of compound **5d** in DMSO-d_6

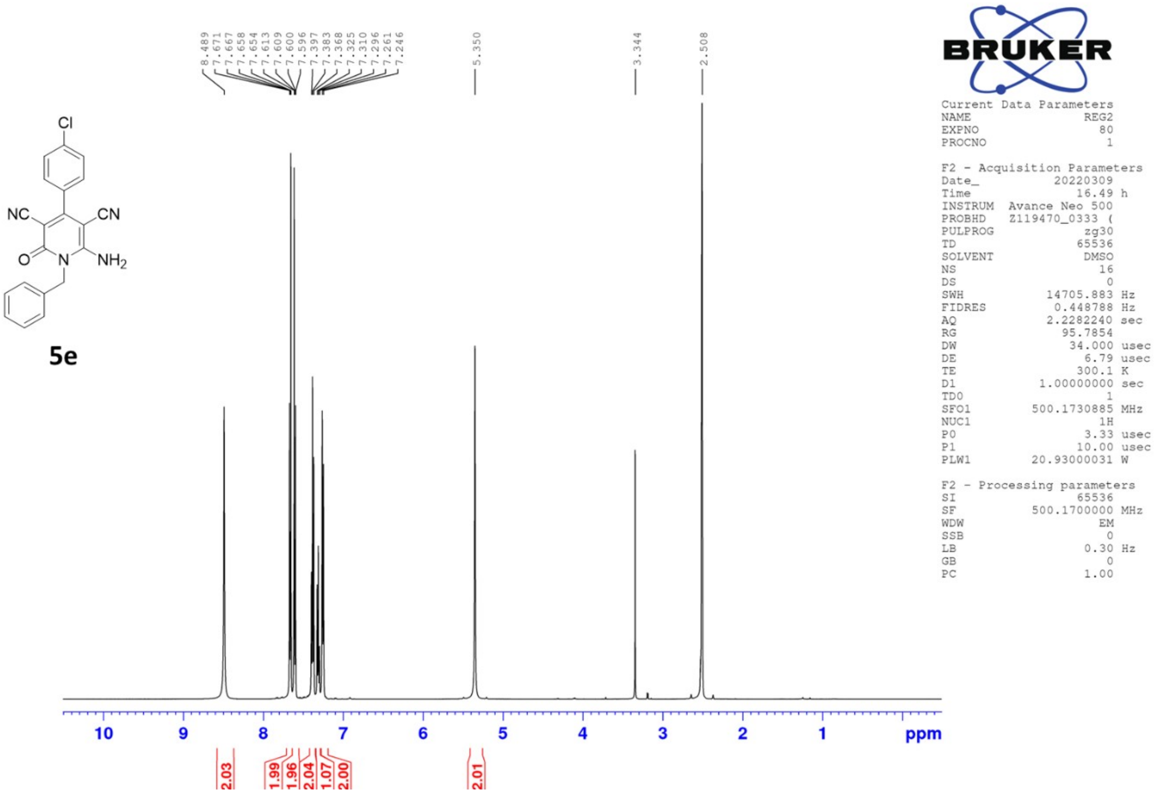

Figure S11. ^{1}H NMR spectrum of compound **5e** at 500 MHz in DMSO-d$_6$

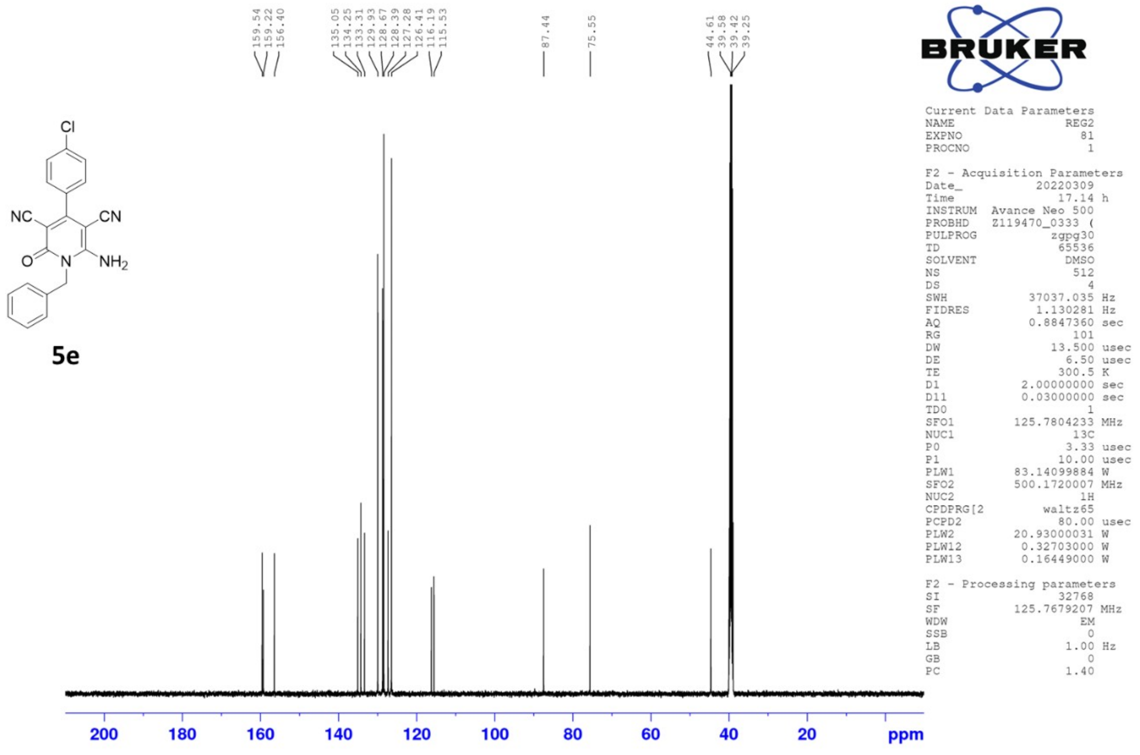

Figure S12. ^{13}C{^{1}H} NMR spectrum of compound **5e** at 125 MHz in DMSO-d$_6$

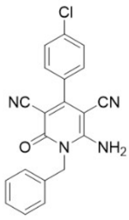

5e

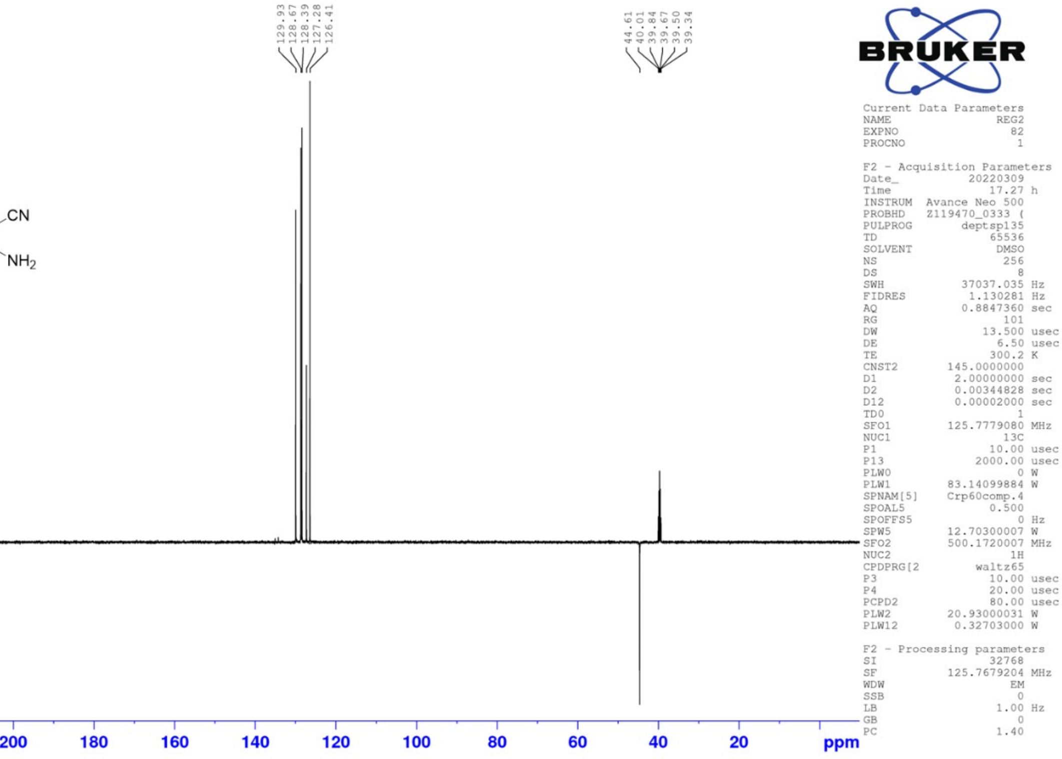

Figure S13. DEPT-135 spectrum of compound **5e** at 125 MHz in DMSO-d$_6$

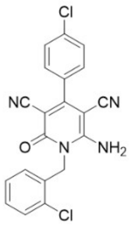

5f

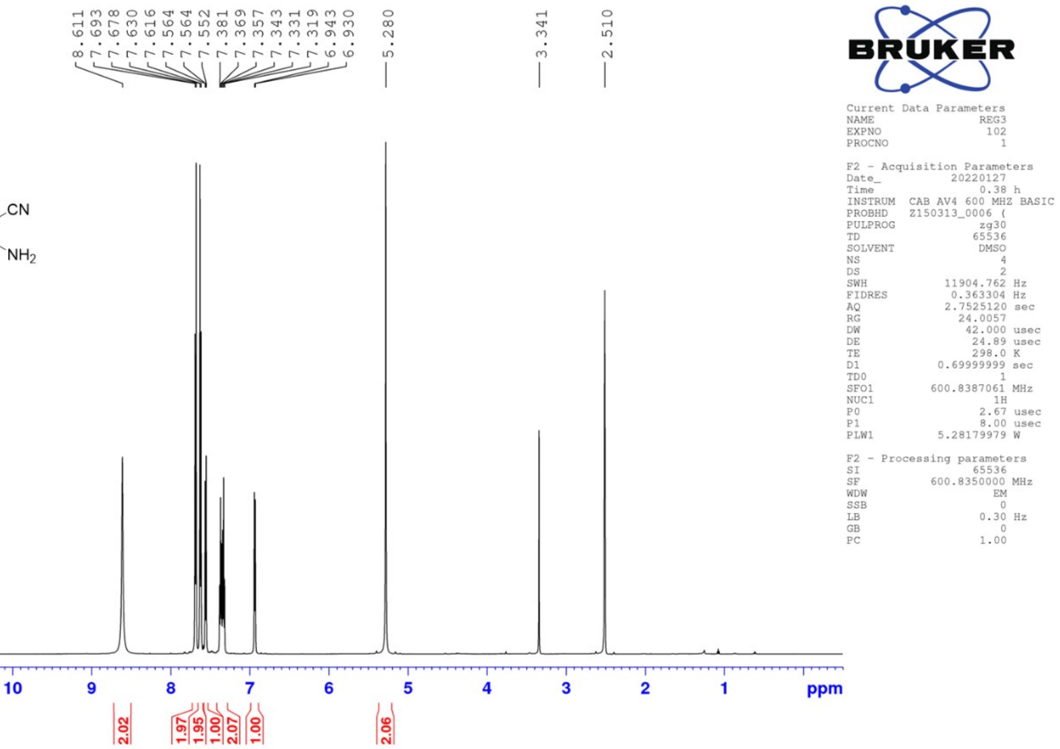

Figure S14. ^{1}H NMR spectrum of compound **5f** at 600 MHz in DMSO-d$_6$

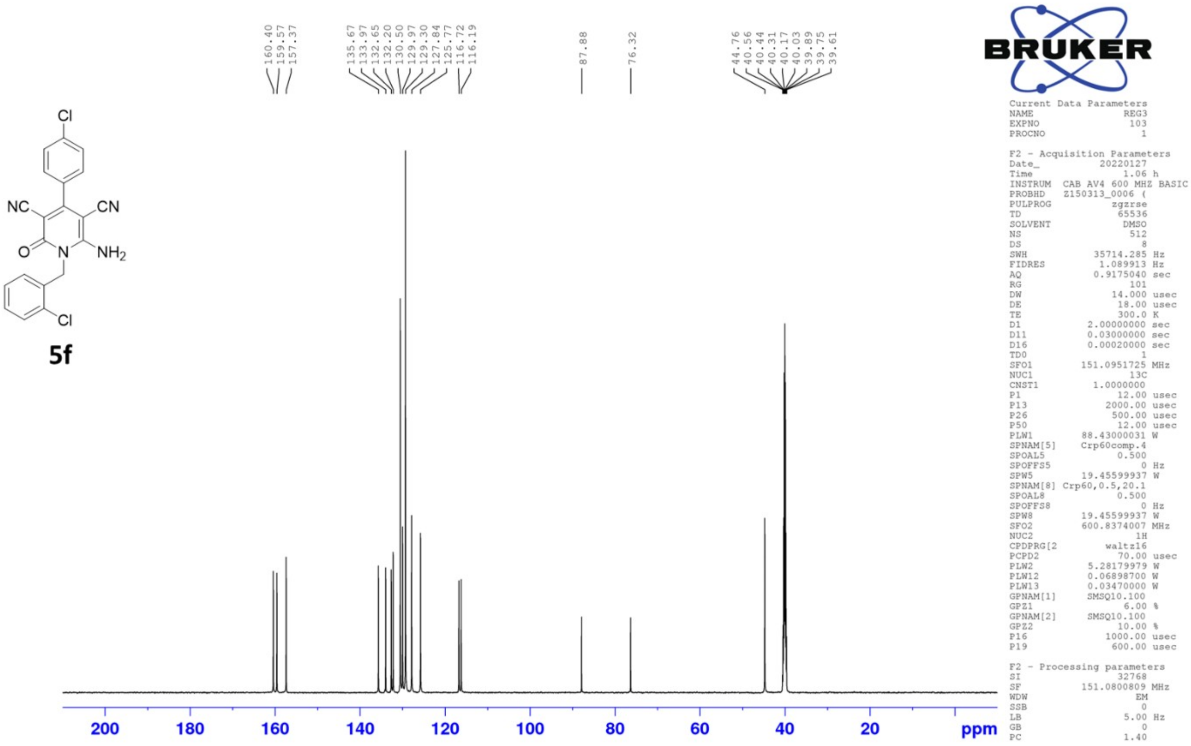

Figure S15. $^{13}C\{^{1}H\}$ NMR spectrum of compound **5f** at 151 MHz in DMSO-d_6

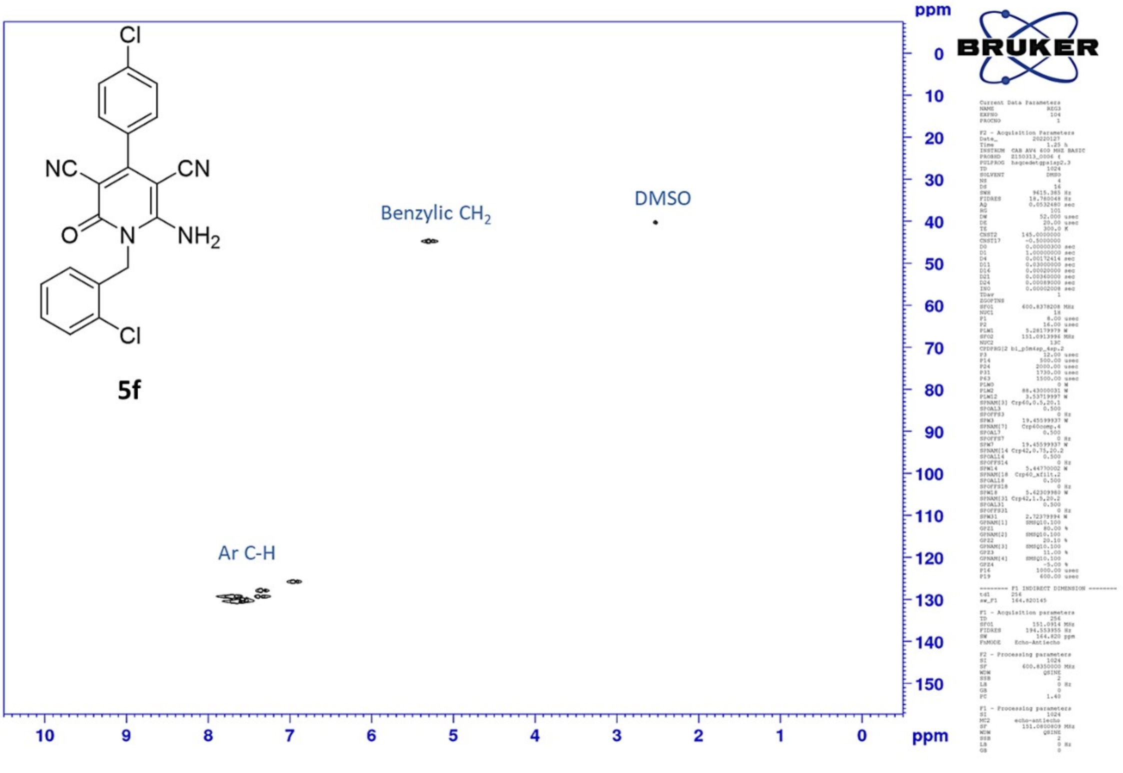

Figure S16. HSQC spectrum of compound **5f** in DMSO-d_6

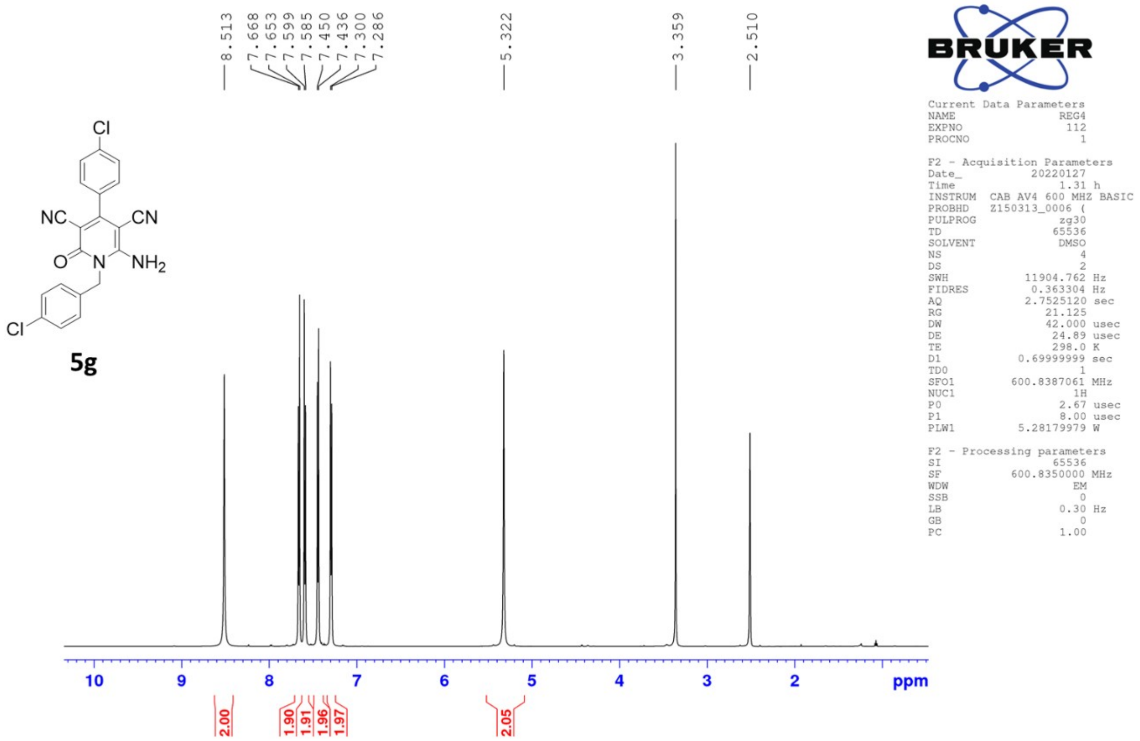

Figure S17. ^{1}H NMR spectrum of compound **5g** at 600 MHz in DMSO-d_6

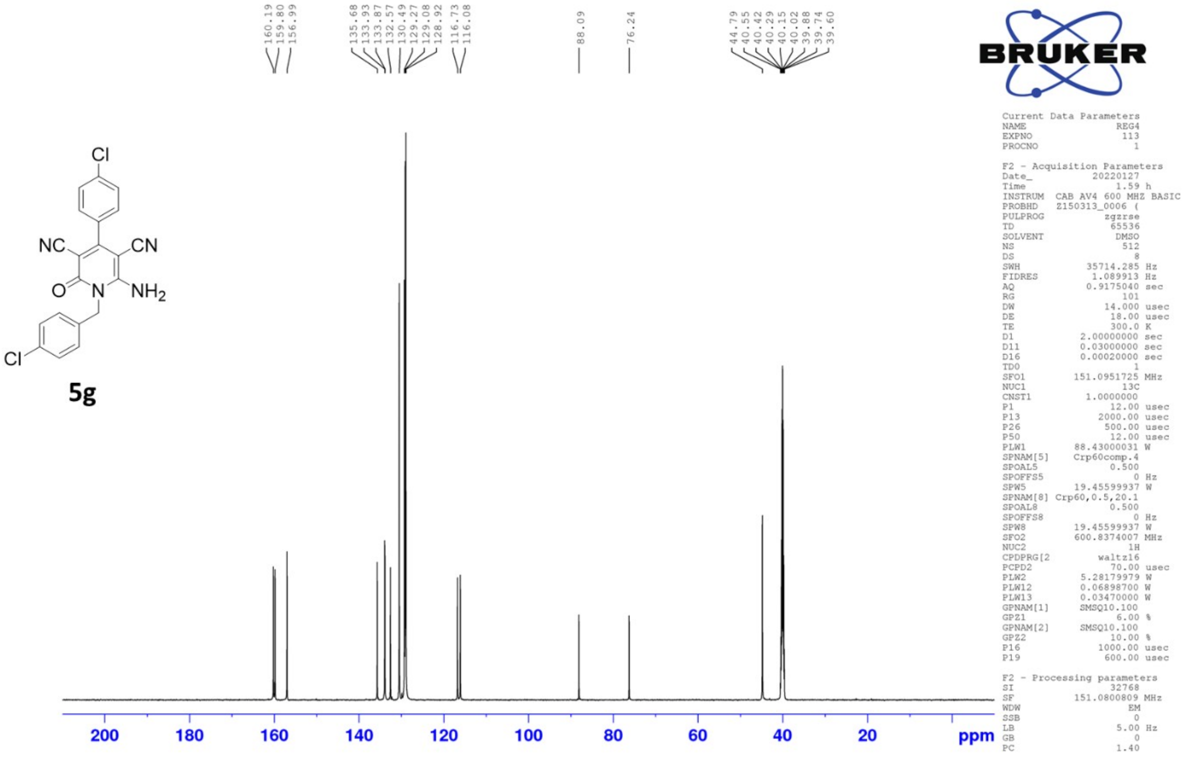

Figure S18. $^{13}C\{^{1}H\}$ NMR spectrum of compound **5g** at 151 MHz in DMSO-d_6

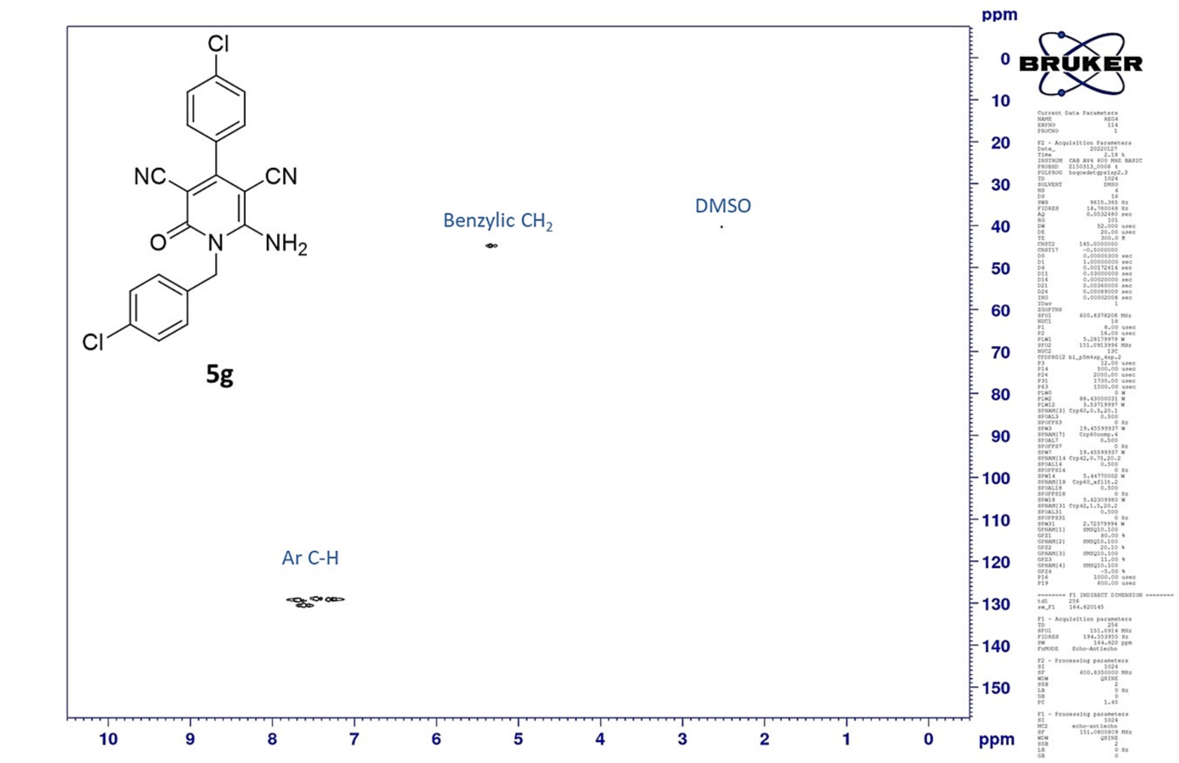

Figure S19. DEPT-135 spectrum of compound **5g** at 125 MHz in DMSO-d$_6$

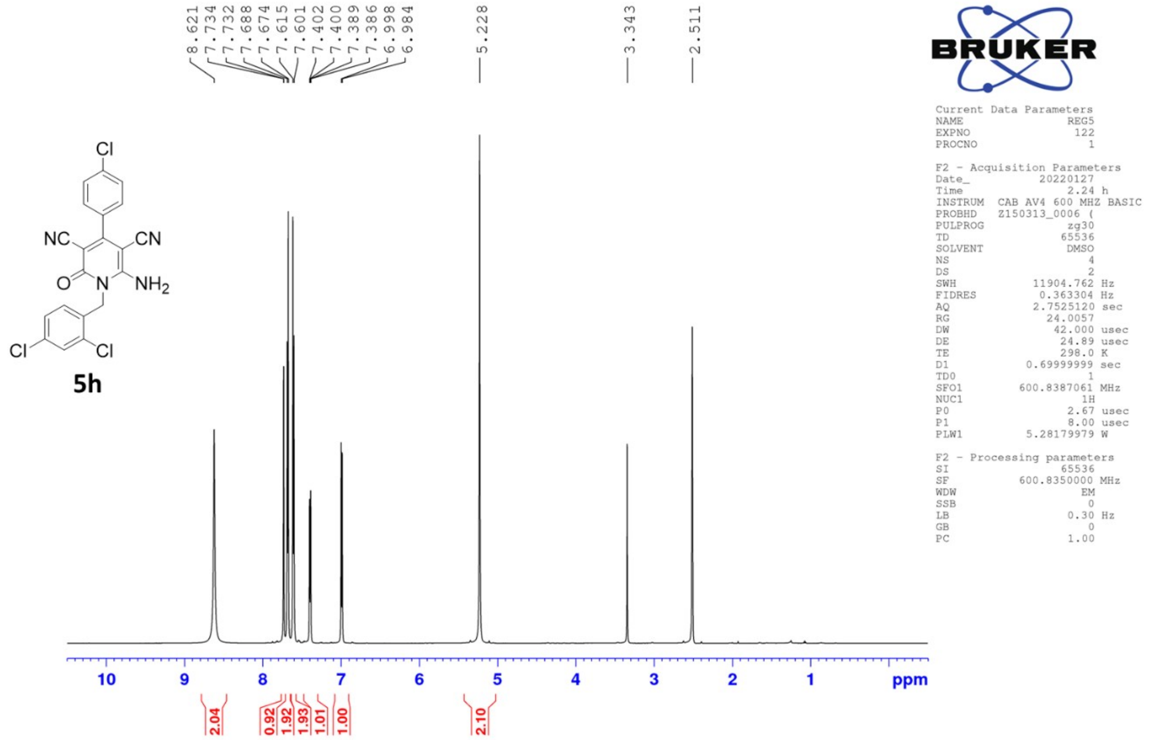

Figure S20. ^{1}H NMR spectrum of compound **5h** at 600 MHz in DMSO-d$_6$

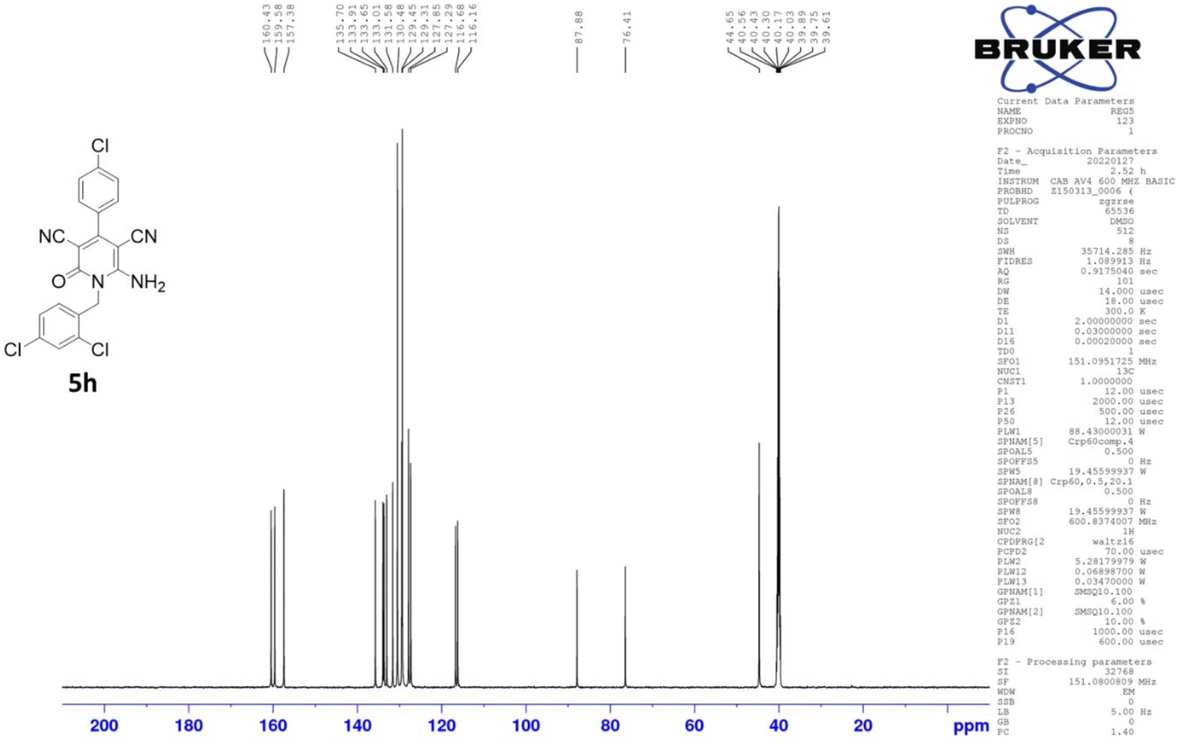

Figure S21. $^{13}C\{^{1}H\}$ NMR spectrum of compound **5h** at 151 MHz in DMSO-d$_6$

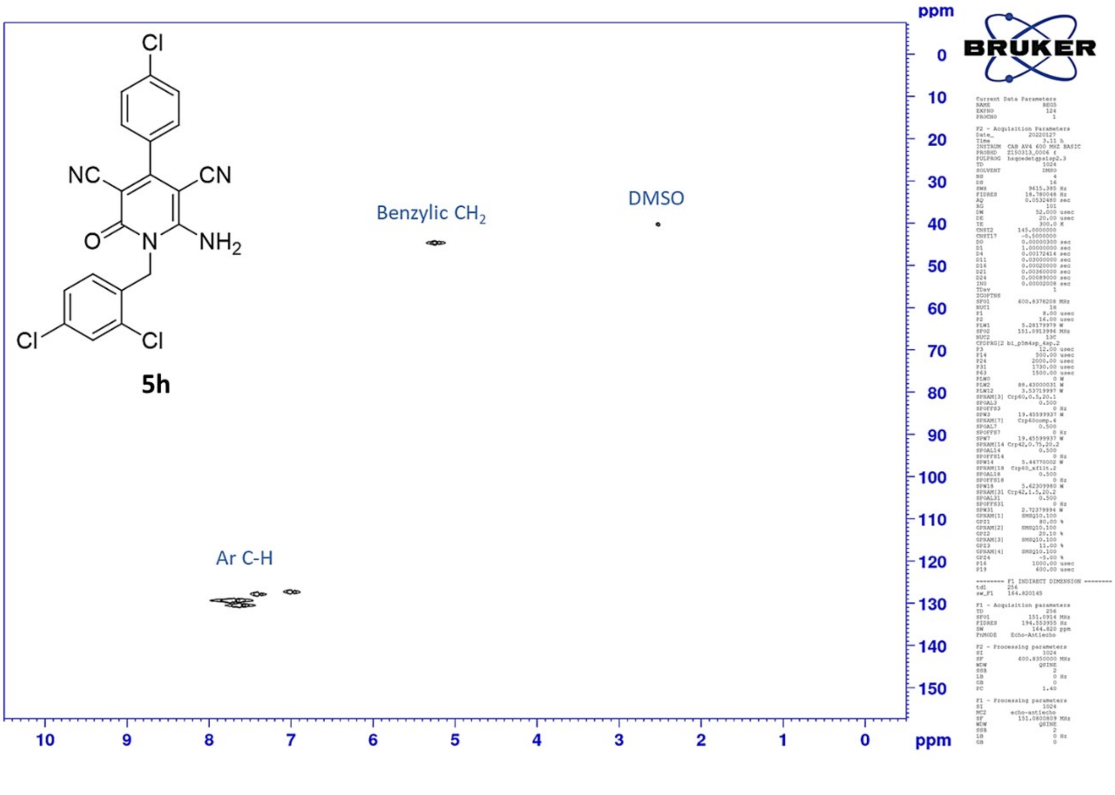

Figure S22. HSQC spectrum of compound **5h** in DMSO-d$_6$

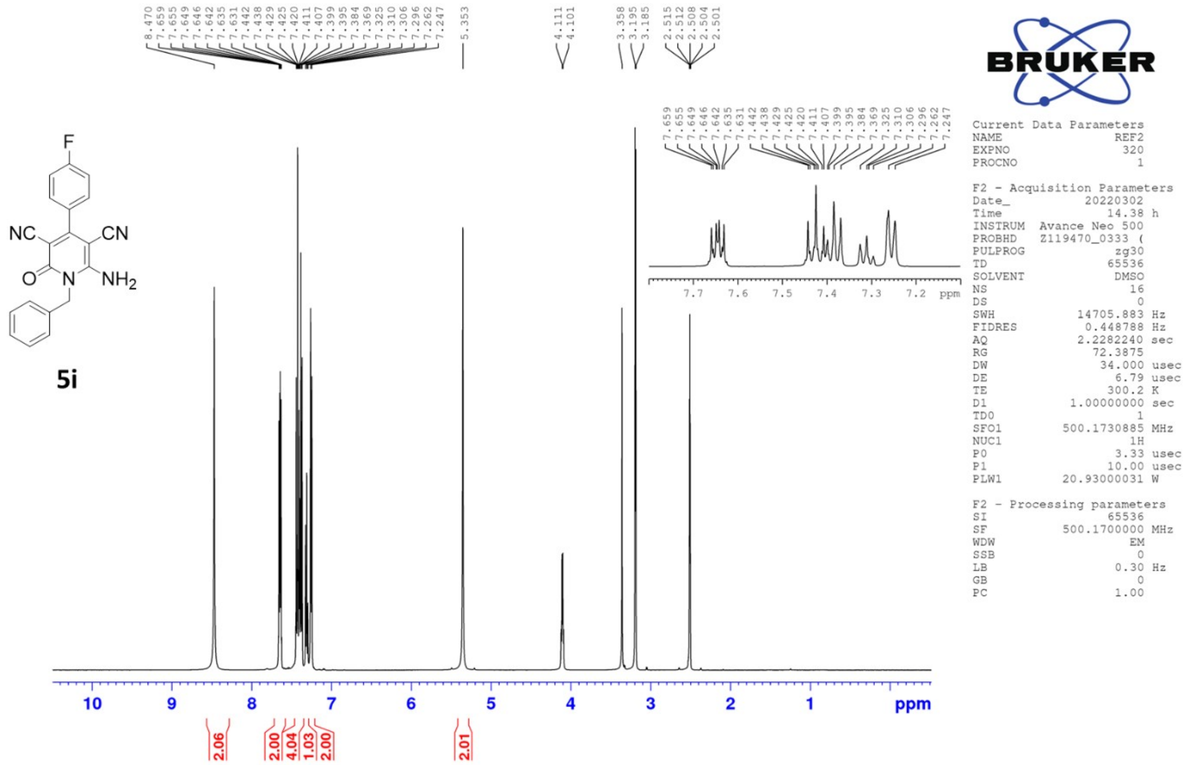

Figure S23. ^{1}H NMR spectrum of compound **5i** at 500 MHz in DMSO-d$_6$

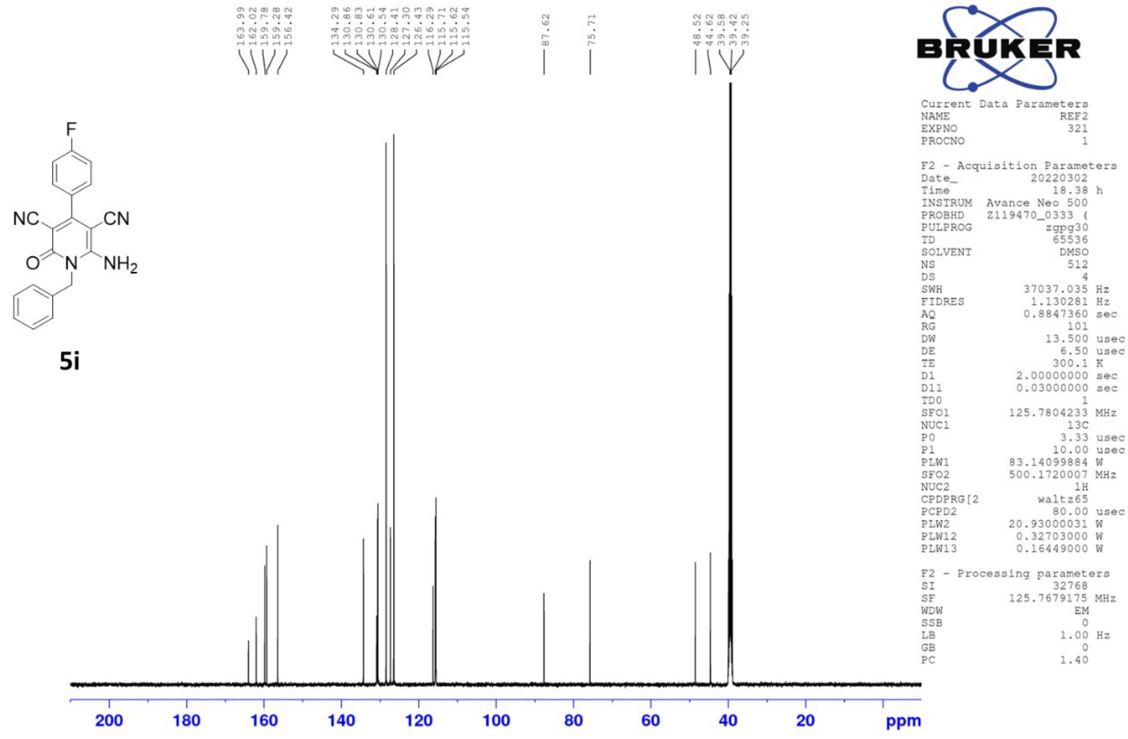

Figure S24. ^{13}C{^{1}H} NMR spectrum of compound **5i** at 125 MHz in DMSO-d$_6$

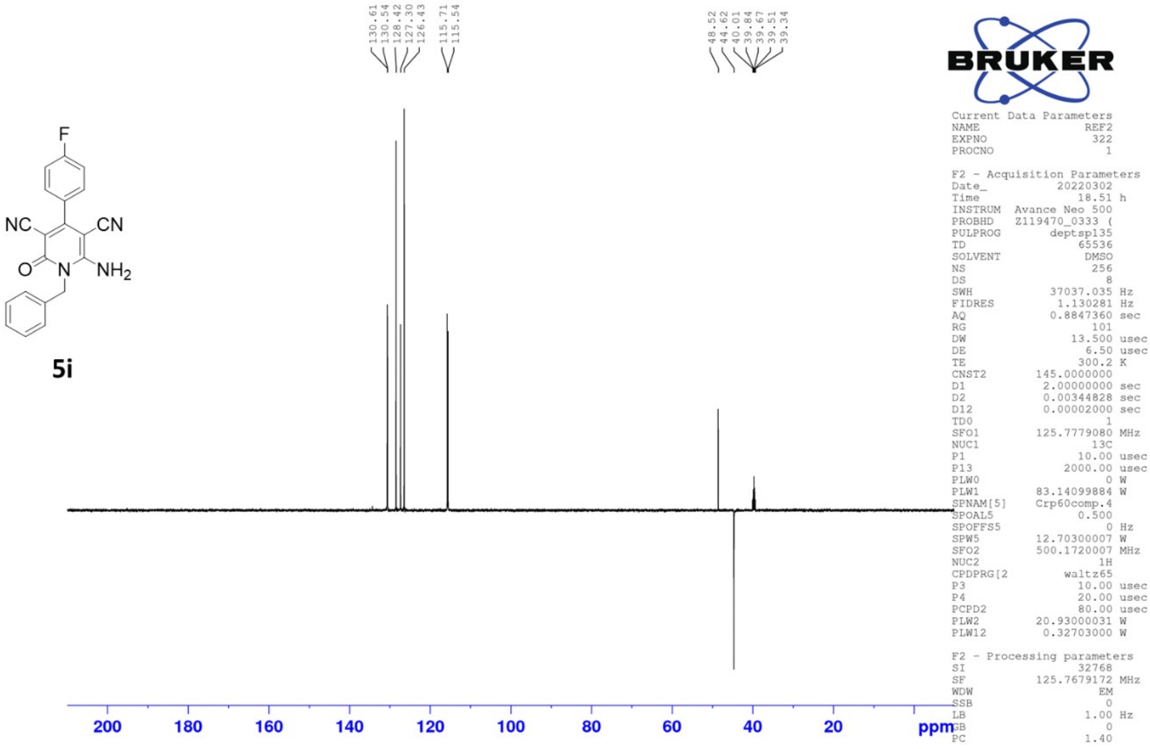

Figure S25. DEPT-135 spectrum of compound **5i** at 125 MHz in DMSO-d$_6$

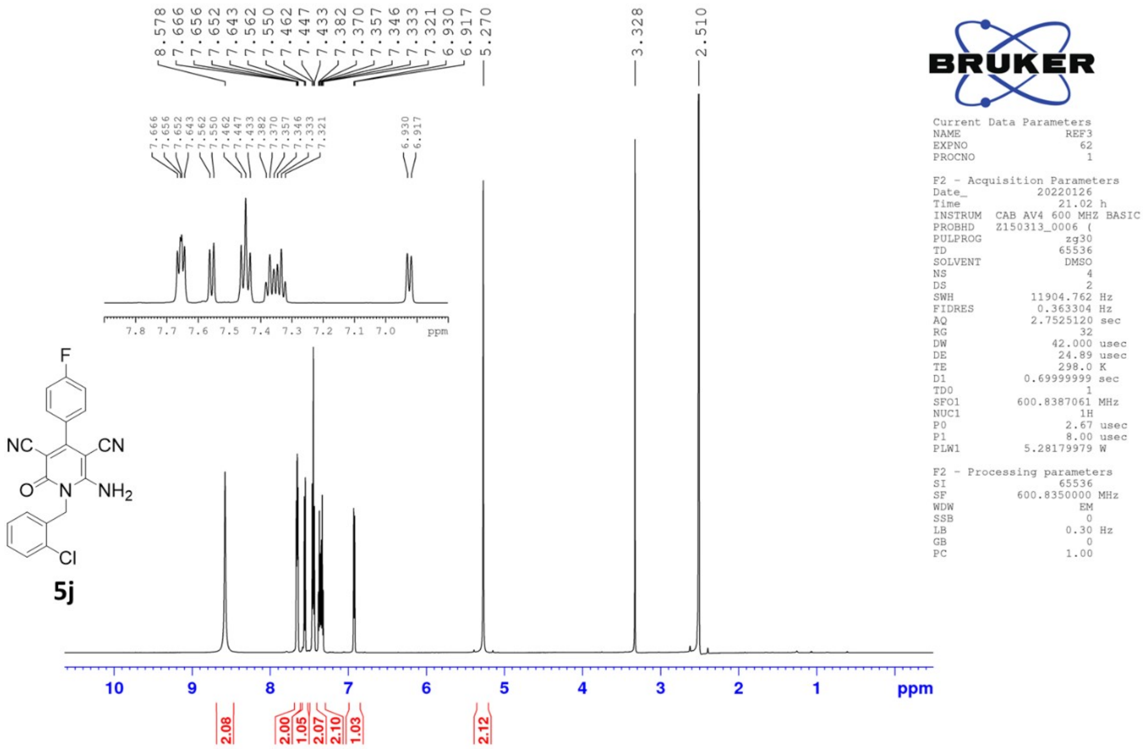

Figure S26. ^{1}H NMR spectrum of compound **5j** at 600 MHz in DMSO-d$_6$

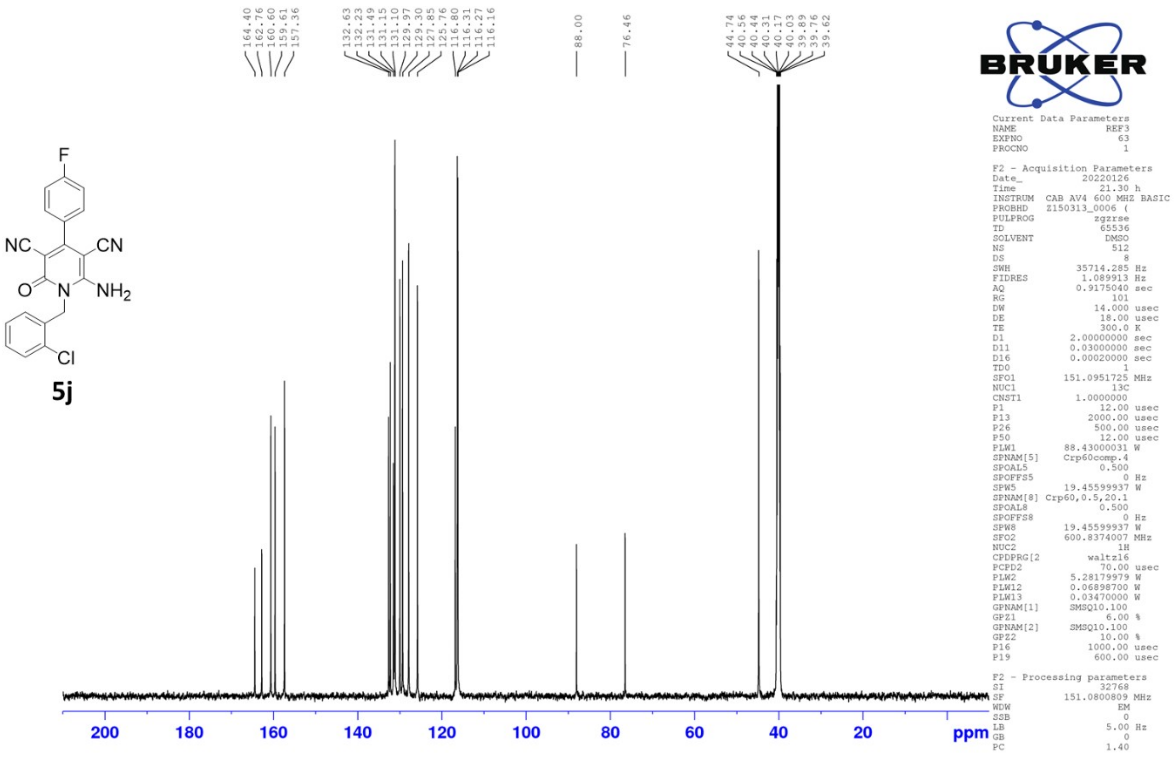

Figure S27. $^{13}C\{^{1}H\}$ NMR spectrum of compound **5j** at 151 MHz in DMSO-d$_6$

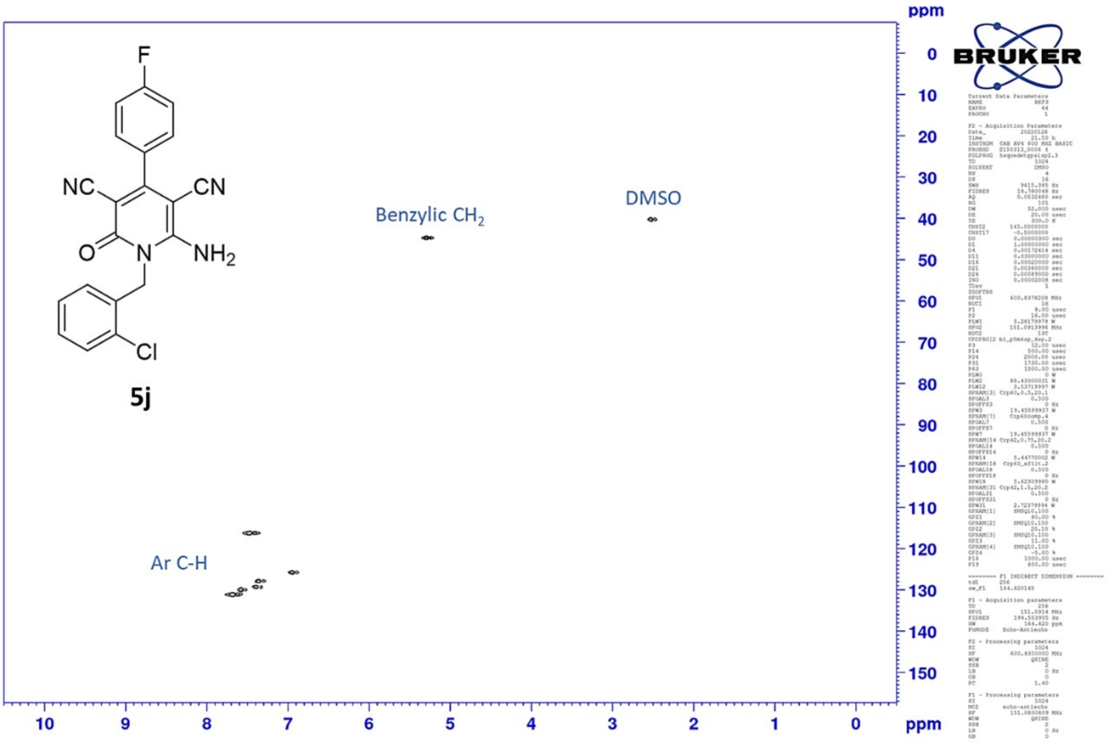

Figure S28. HSQC spectrum of compound **5j** in DMSO-d$_6$

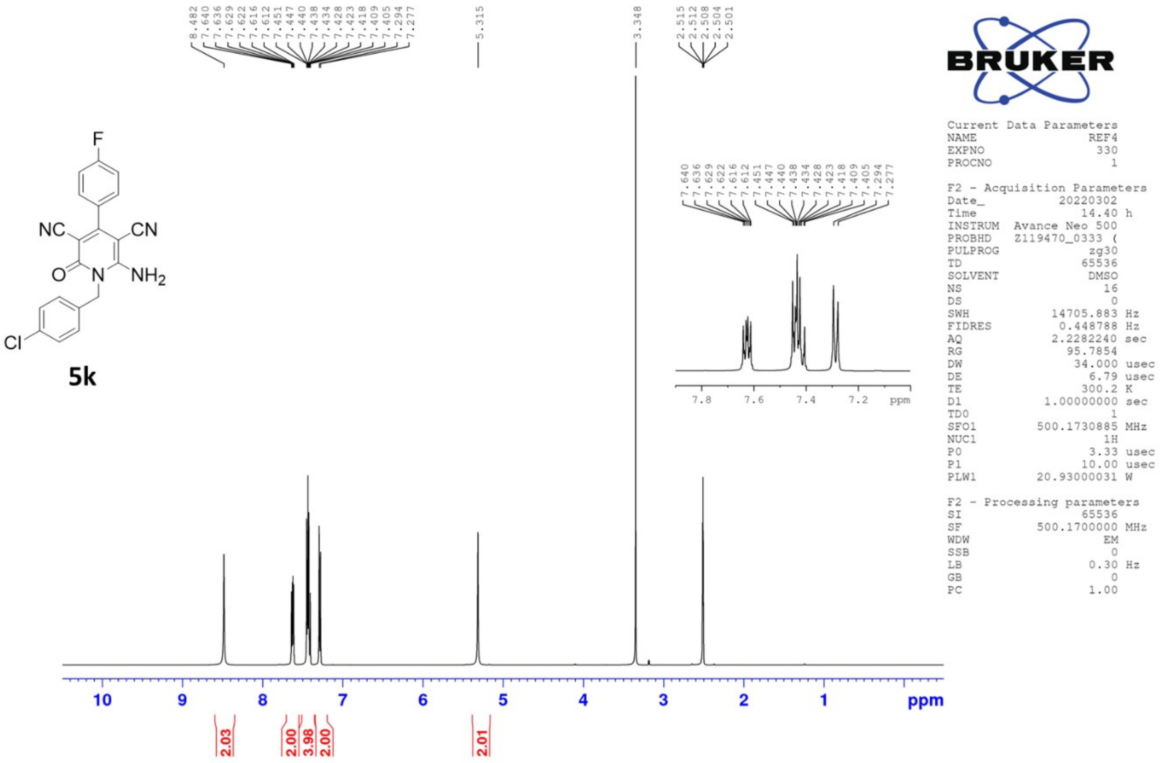

Figure S29. ^{1}H NMR spectrum of compound **5k** at 600 MHz in DMSO-d$_6$

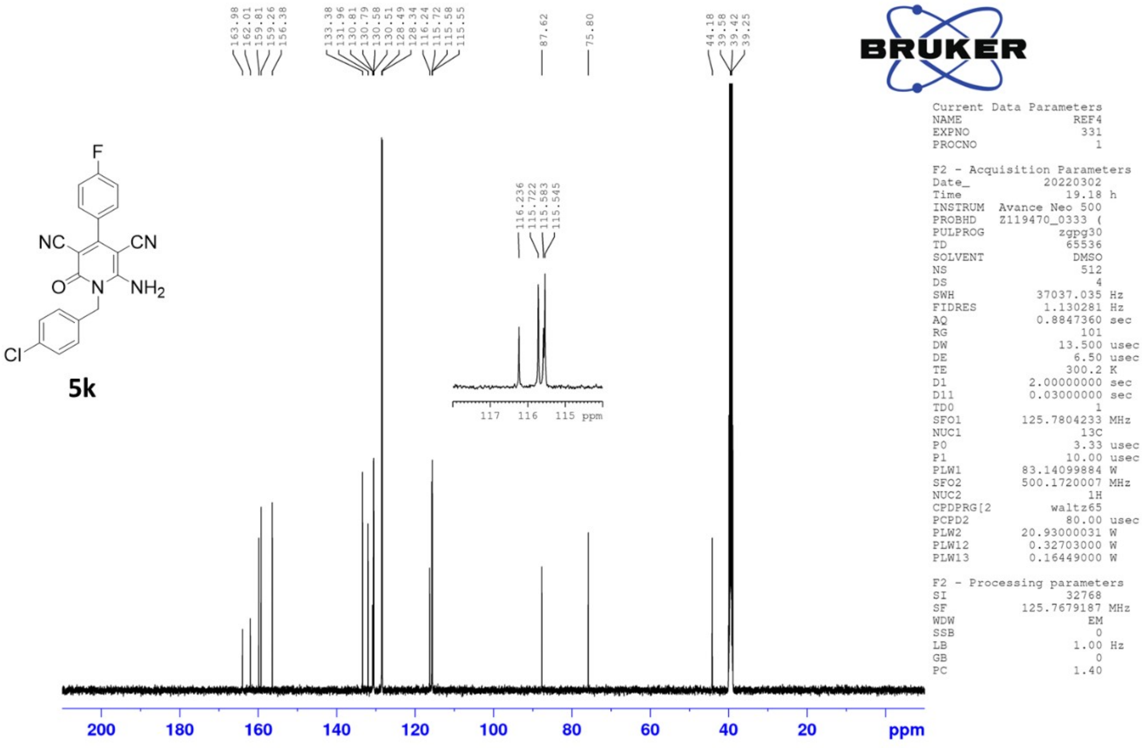

Figure S30. ^{13}C{^{1}H} NMR spectrum of compound **5k** at 151 MHz in DMSO-d$_6$

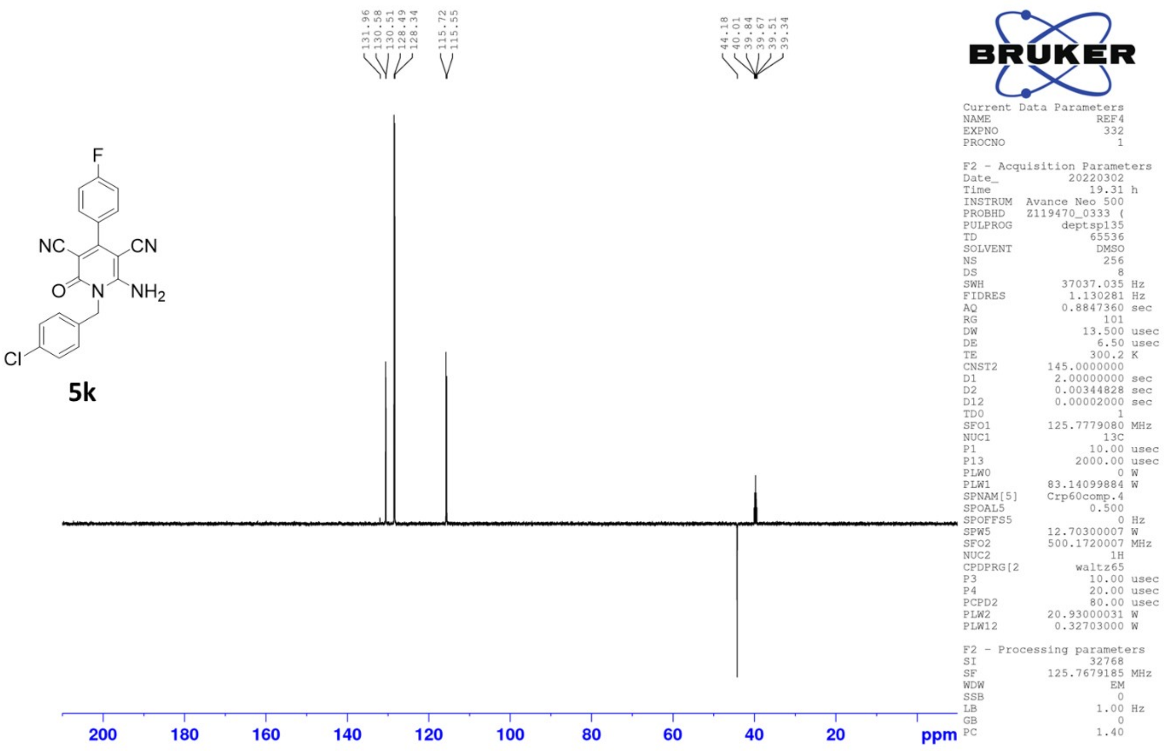

Figure S31. DEPT-135 spectrum of compound **5k** at 125 MHz in DMSO-d_6

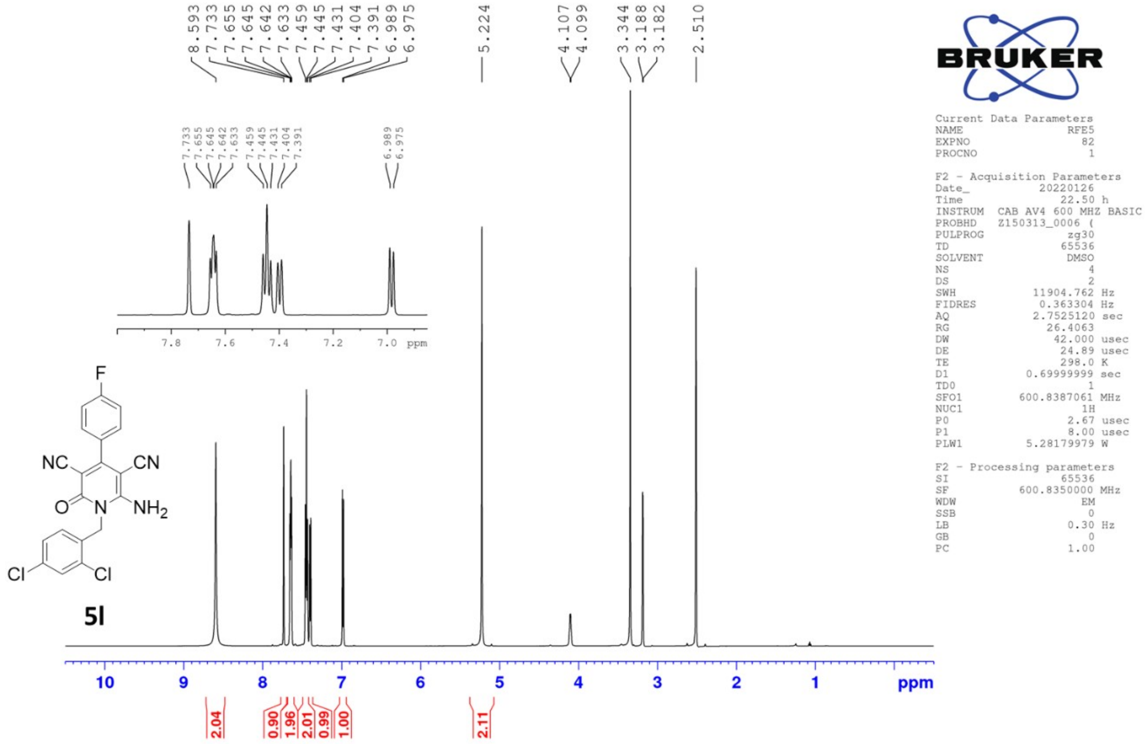

Figure S32. ^1H NMR spectrum of compound **5l** at 600 MHz in DMSO-d_6

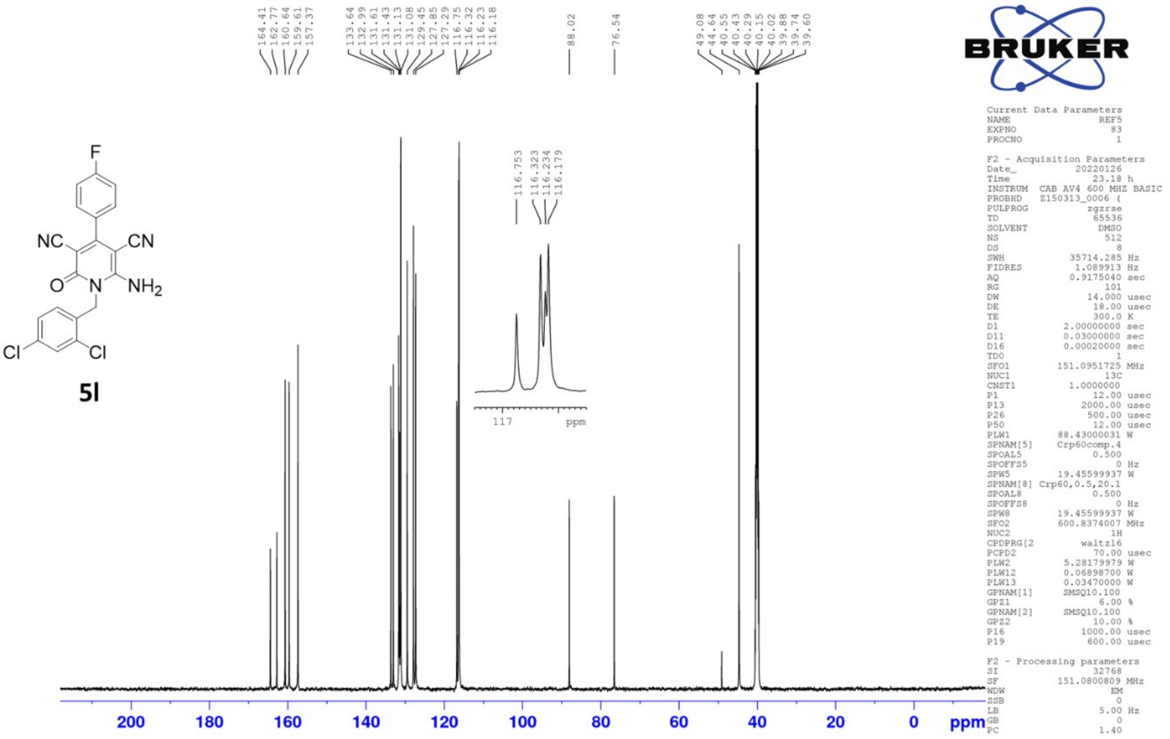

Figure S33. $^{13}C\{^{1}H\}$ NMR spectrum of compound **5l** at 151 MHz in DMSO-d_6

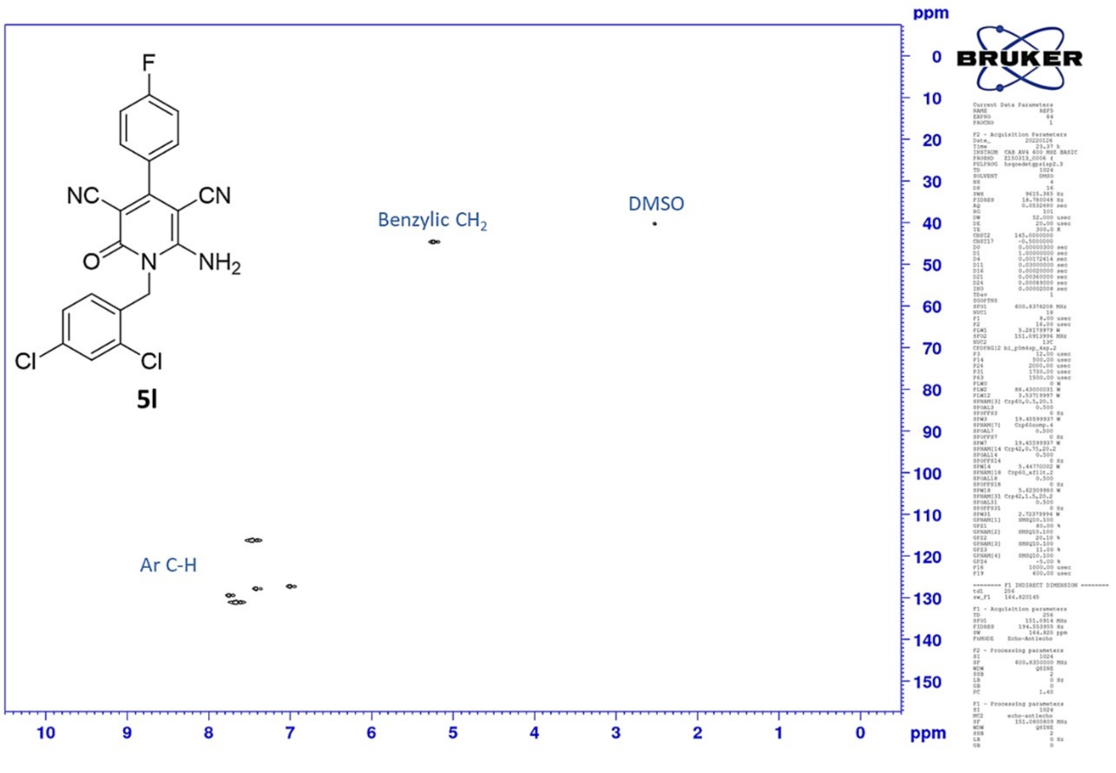

Figure S34. HSQC spectrum of compound **5l** in DMSO-d_6

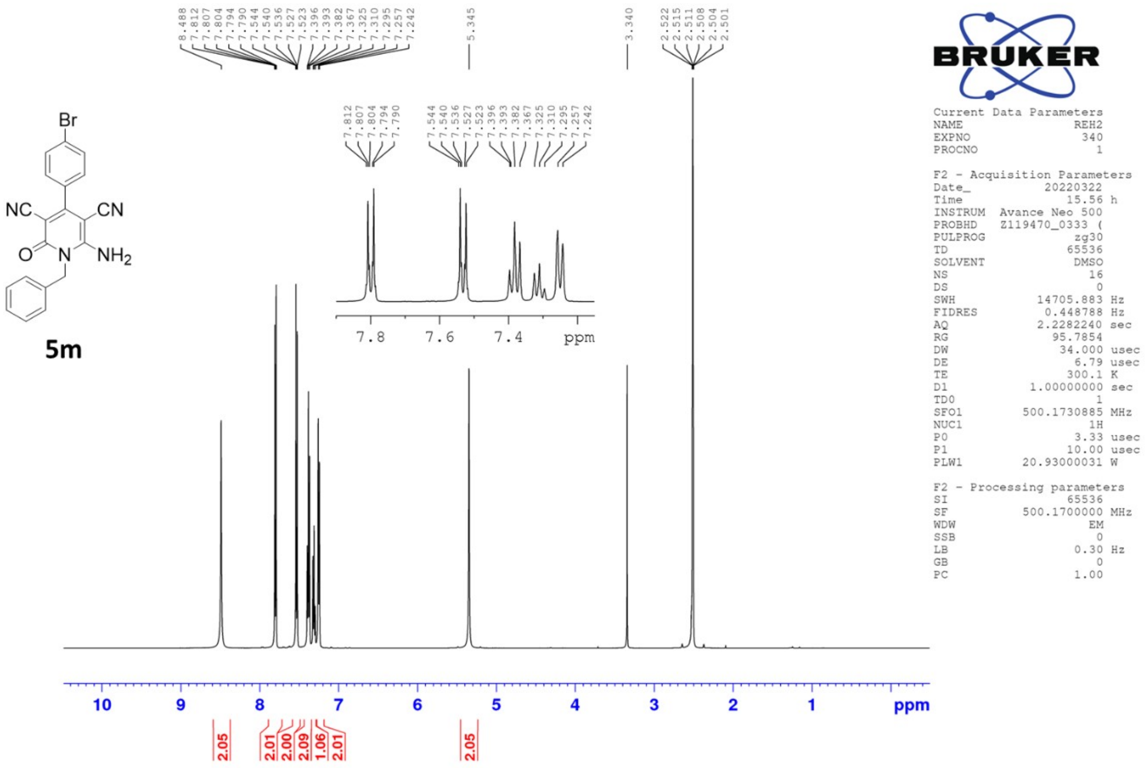

Figure S35. ^{1}H NMR spectrum of compound **5m** at 500 MHz in DMSO-d$_6$

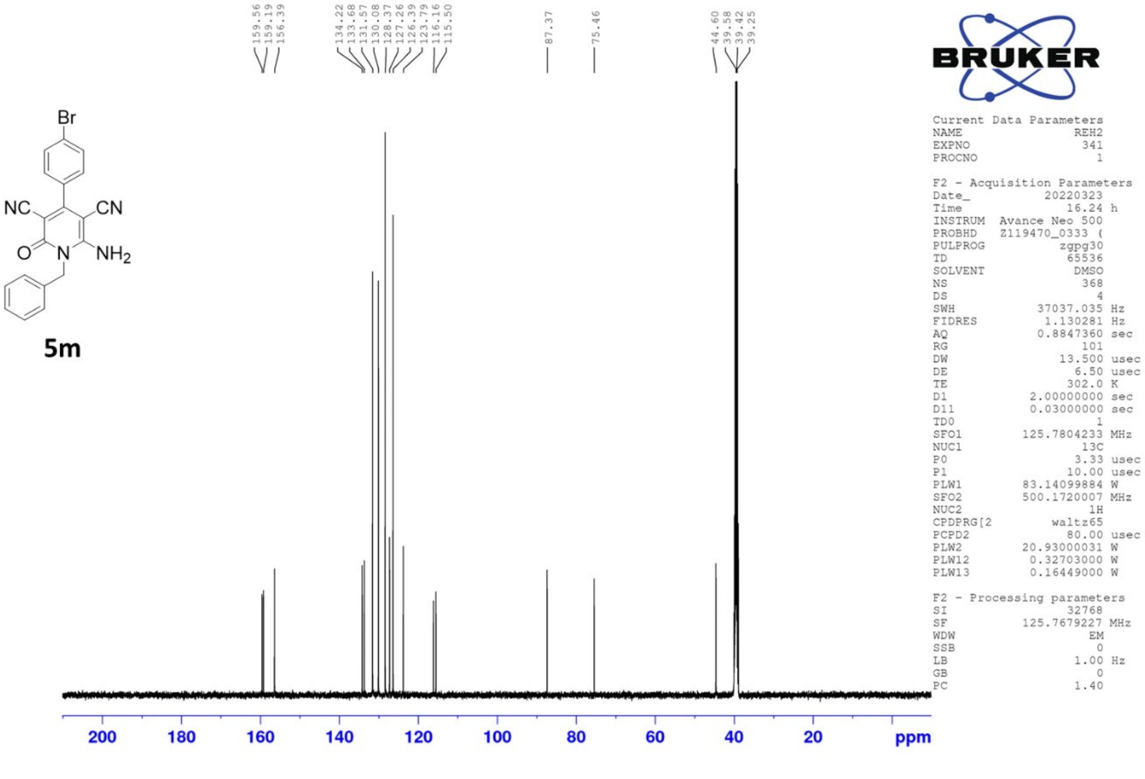

Figure S36. $^{13}C\{^{1}H\}$ NMR spectrum of compound **5m** at 151 MHz in DMSO-d$_6$

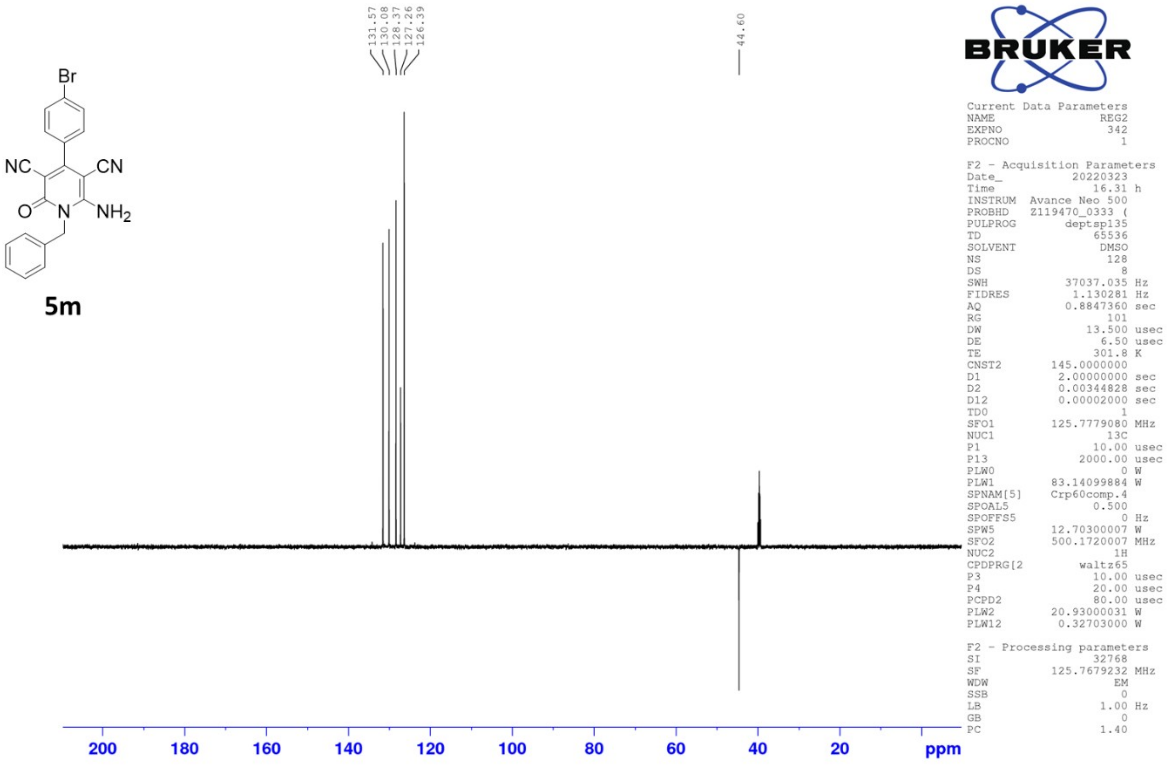

Figure S37. DEPT-135 spectrum of compound **5m** at 125 MHz in DMSO-d$_6$

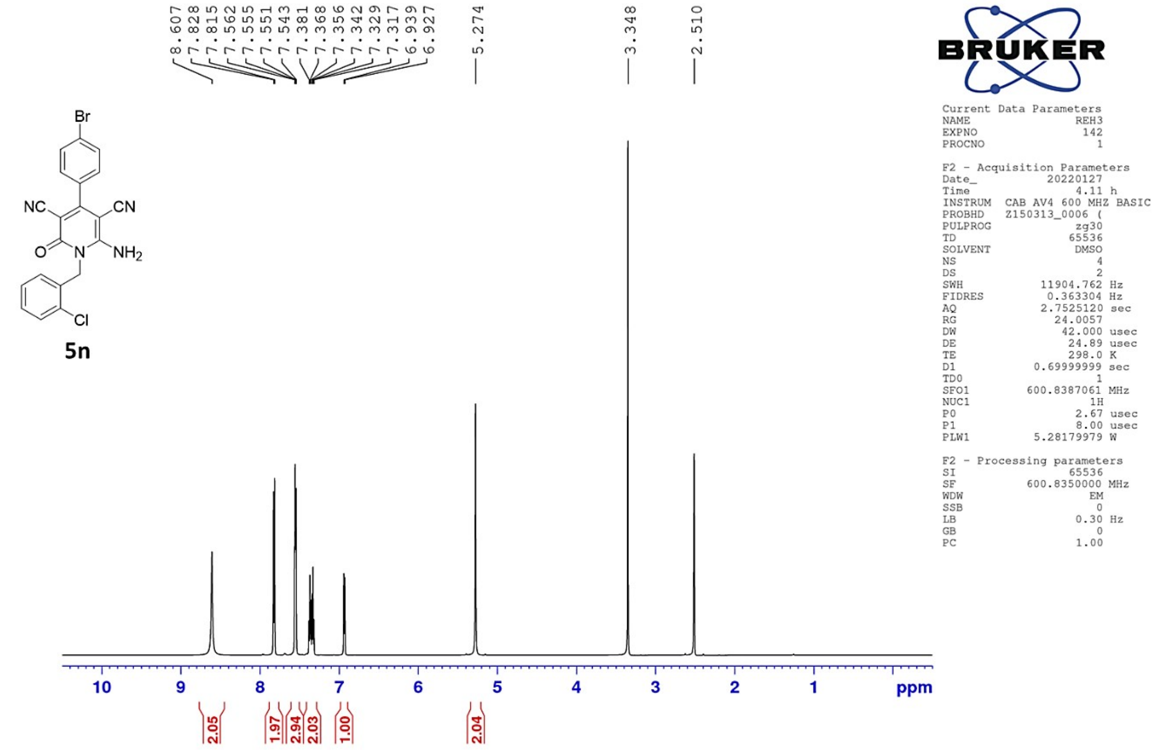

Figure S38. ^{1}H NMR spectrum of compound **5n** at 600 MHz in DMSO-d$_6$

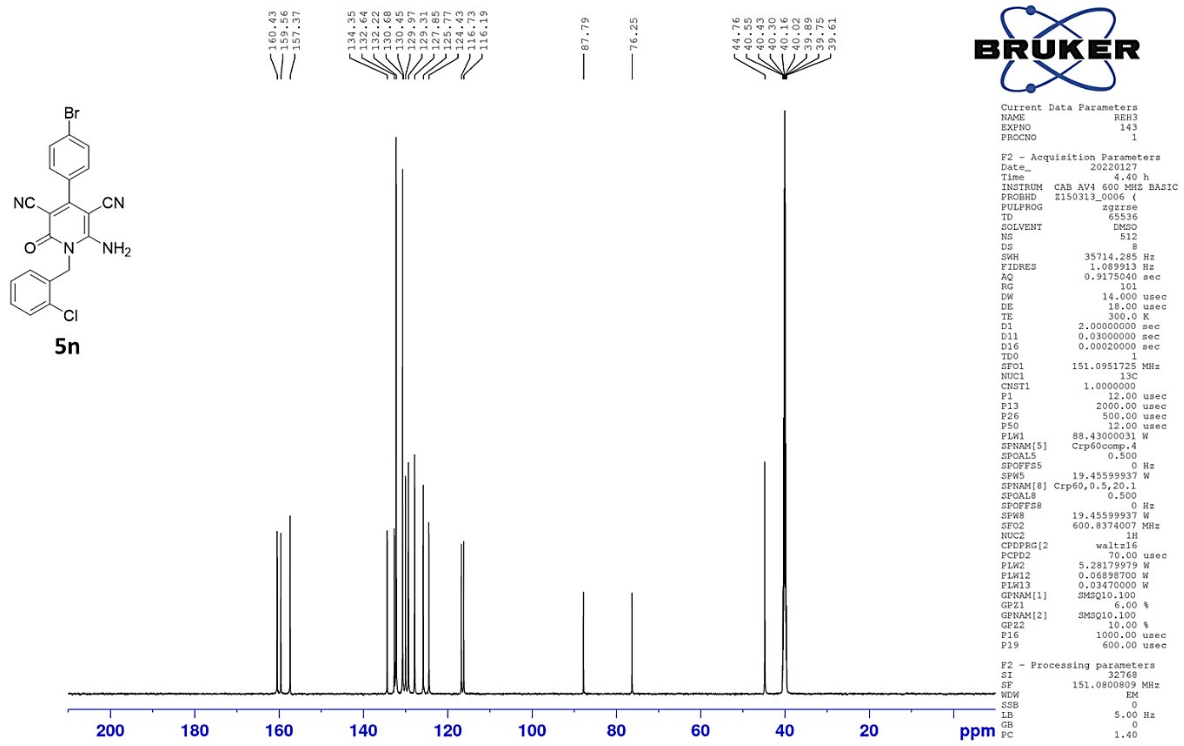

Figure S39. $^{13}C\{^1H\}$ NMR spectrum of compound **5n** at 151 MHz in DMSO-d_6

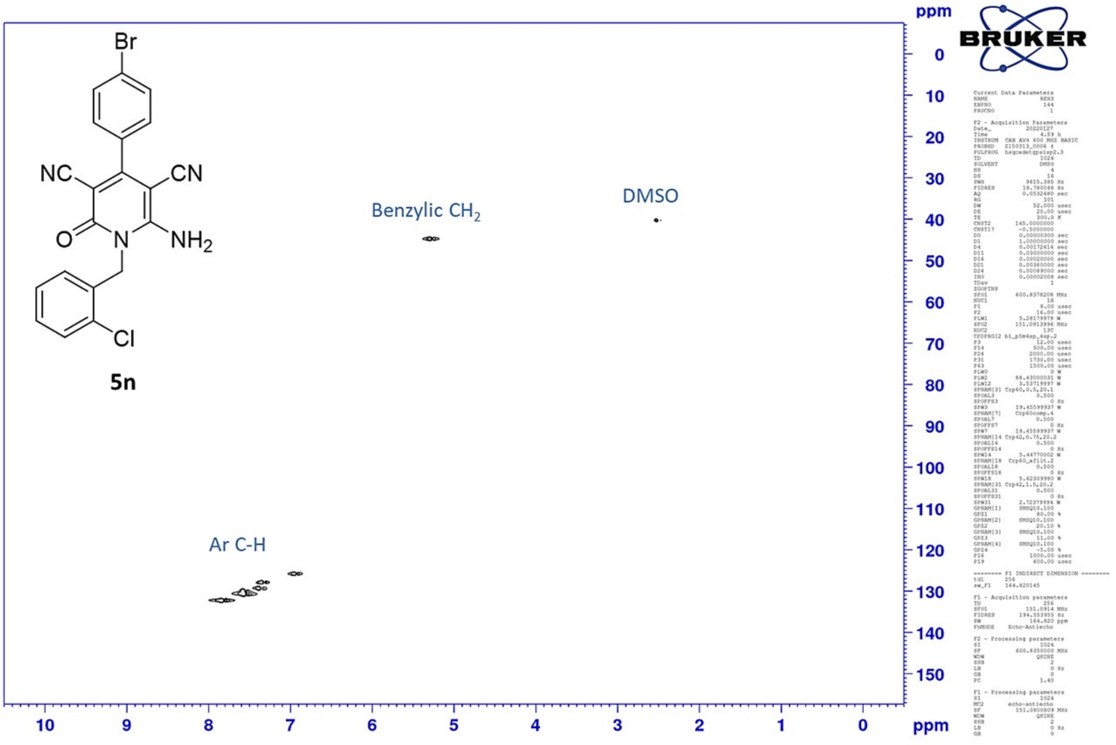

Figure S40. HSQC spectrum of compound **5n** in DMSO-d_6

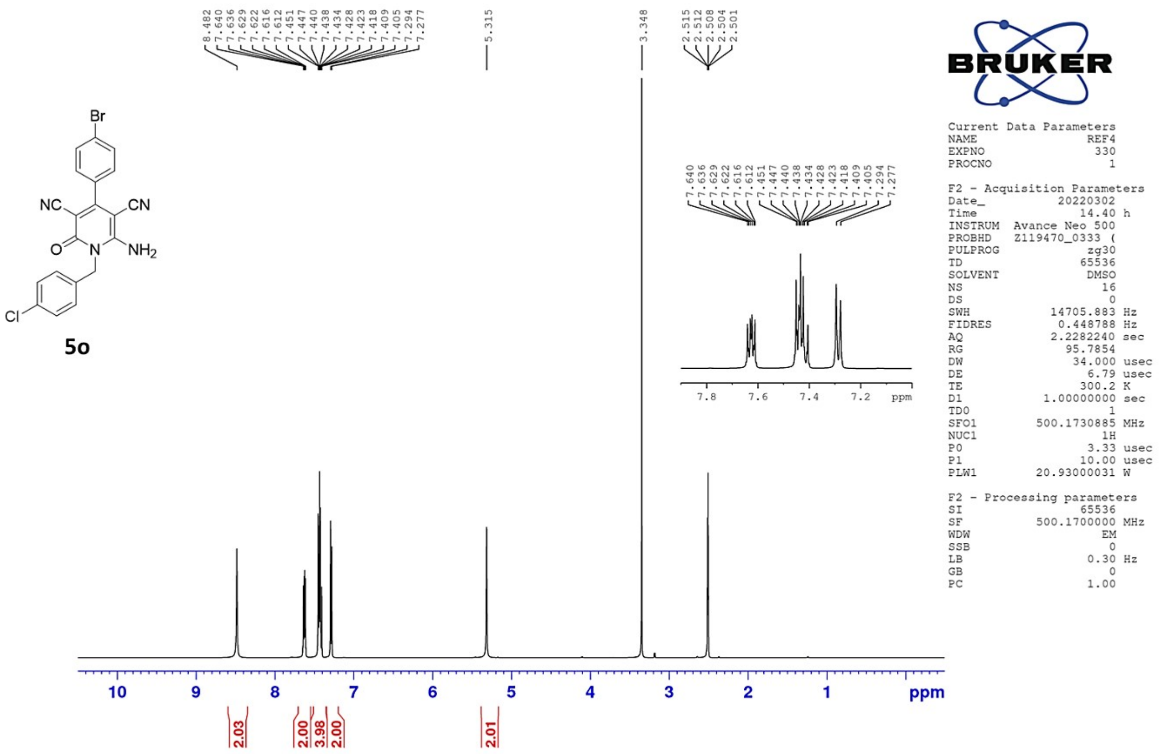

Figure S41. ^{1}H NMR spectrum of compound **5o** at 600 MHz in DMSO-d_6

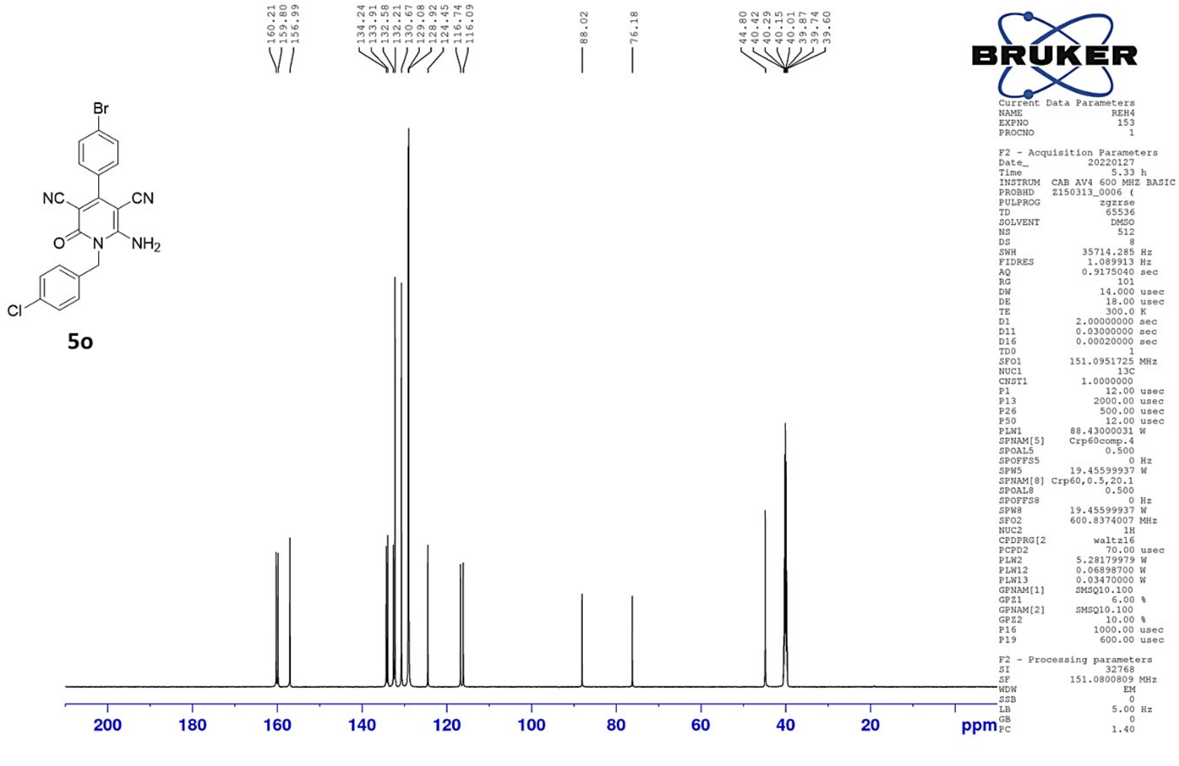

Figure S42. $^{13}C\{^{1}H\}$ NMR spectrum of compound **5o** at 151 MHz in DMSO-d_6

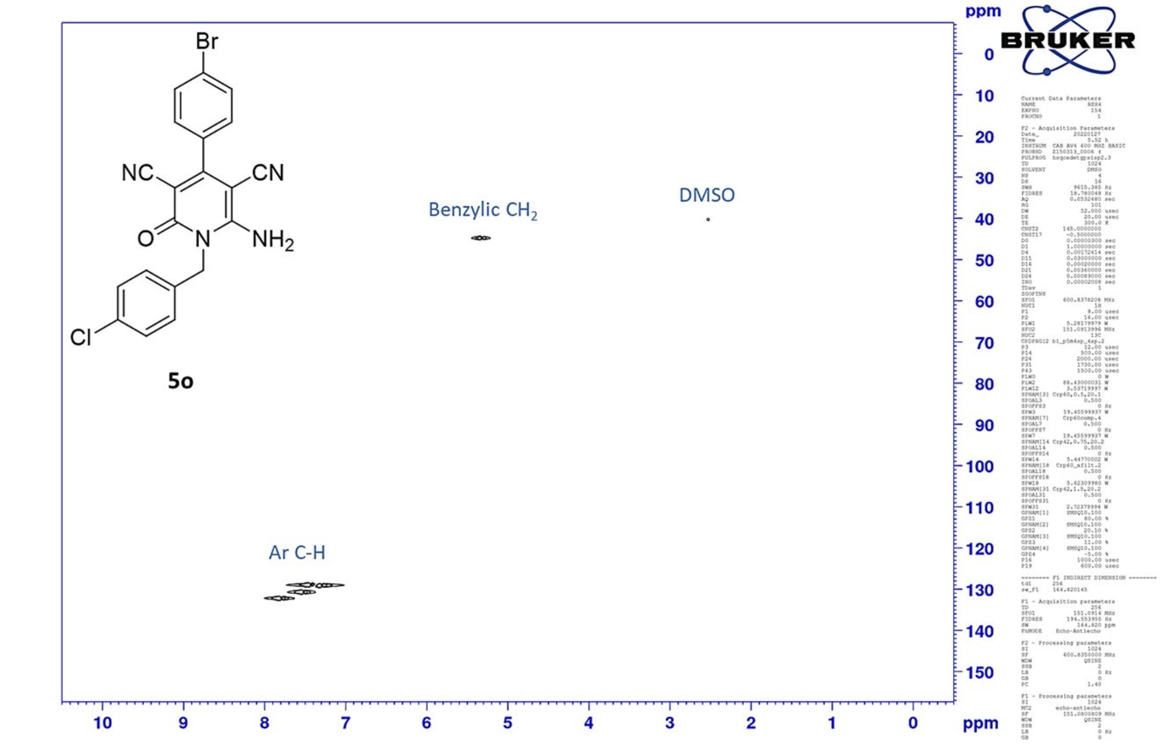

Figure S43. HSQC spectrum of compound **5o** in DMSO-d_6

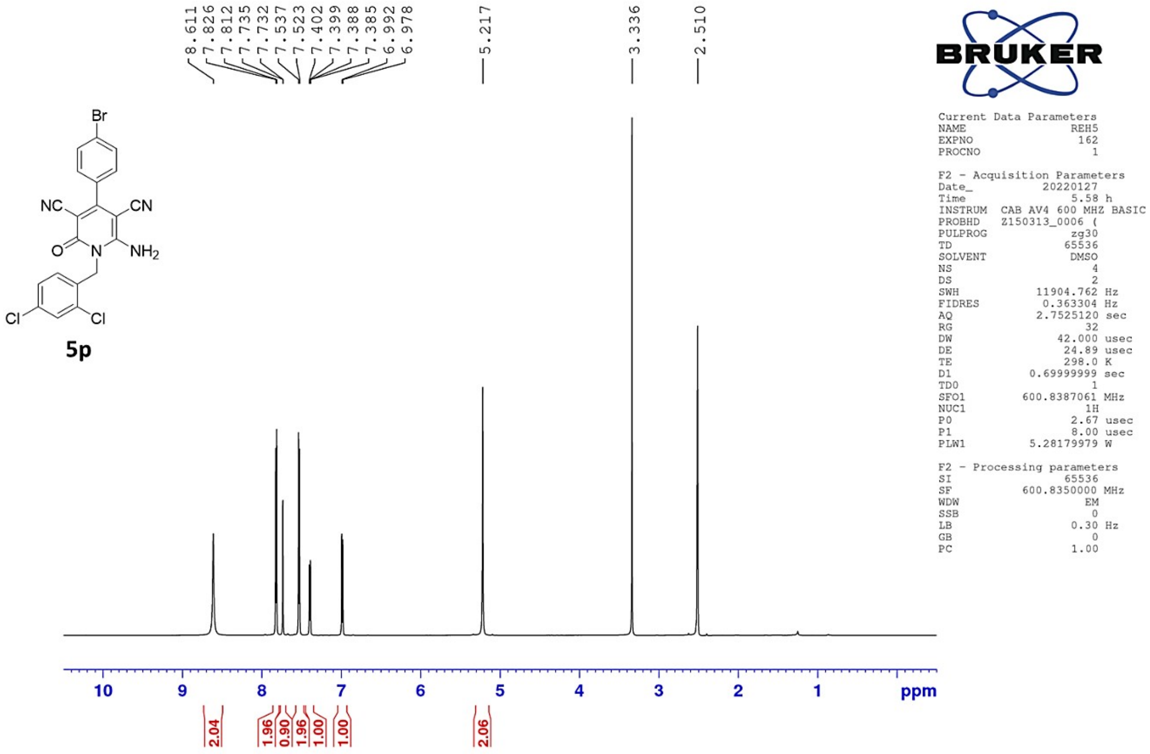

Figure S44. ^{1}H NMR spectrum of compound **5p** at 600 MHz in DMSO-d_6

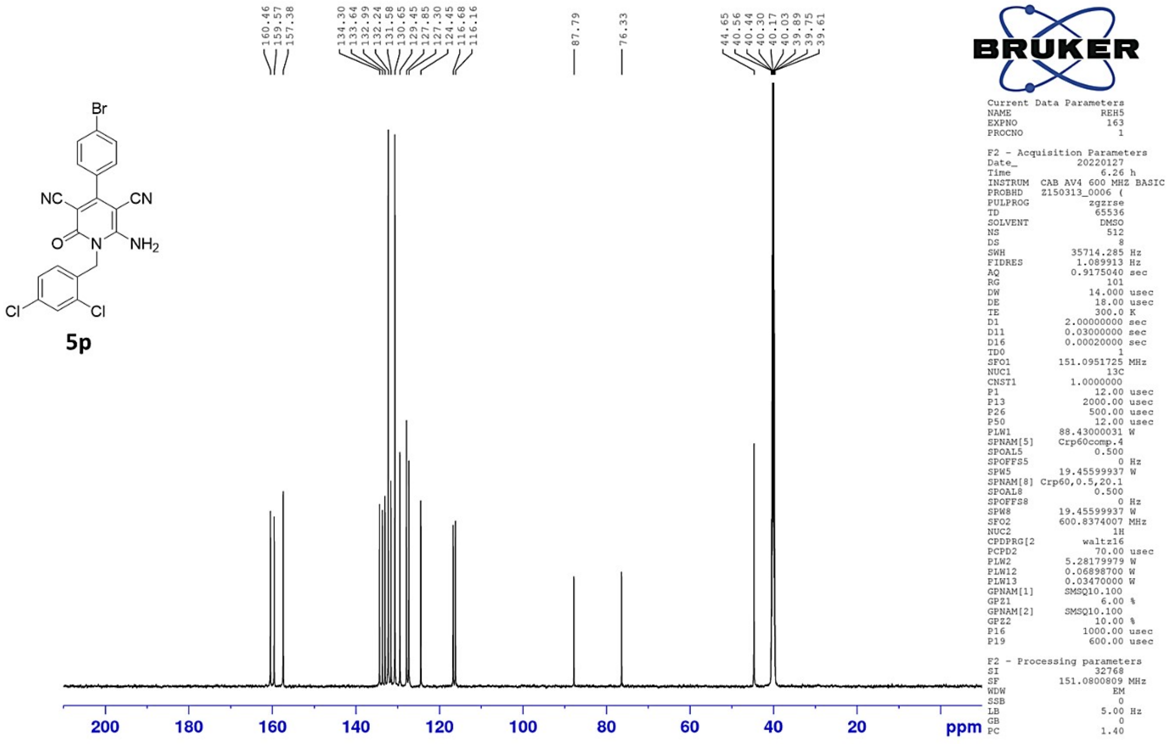

Figure S45. $^{13}C\{^{1}H\}$ NMR spectrum of compound **5p** at 151 MHz in DMSO-d$_6$

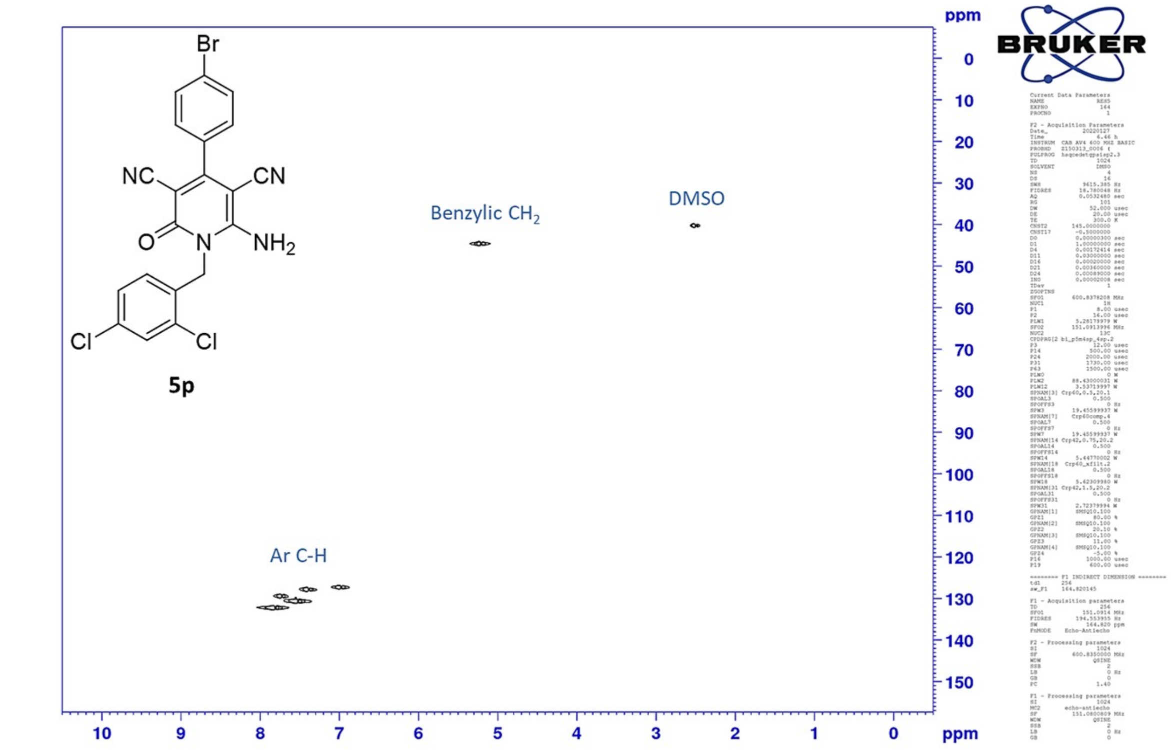

Figure S46. HSQC spectrum of compound **5p** in DMSO-d$_6$

2. Copies of HRMS of **5(a-p)**

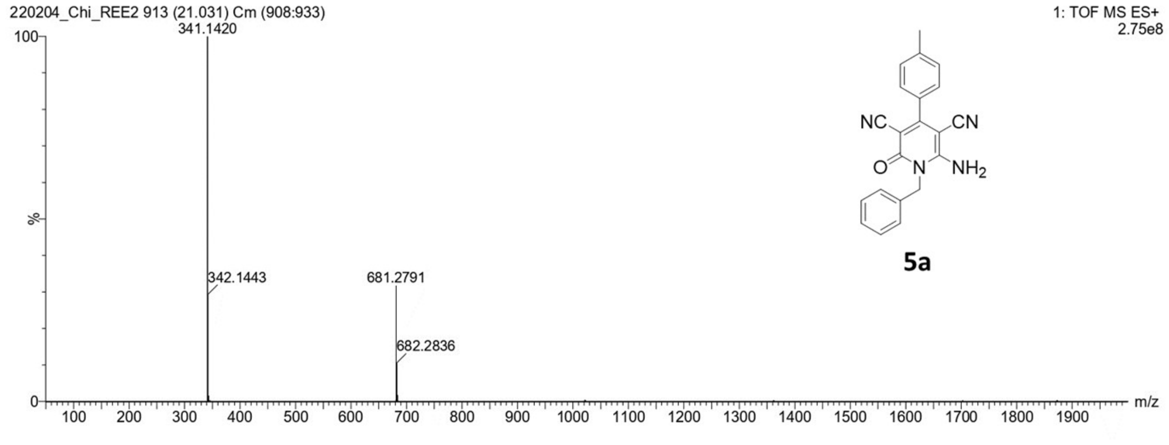

Figure S47. HRMS of compound **5a**.

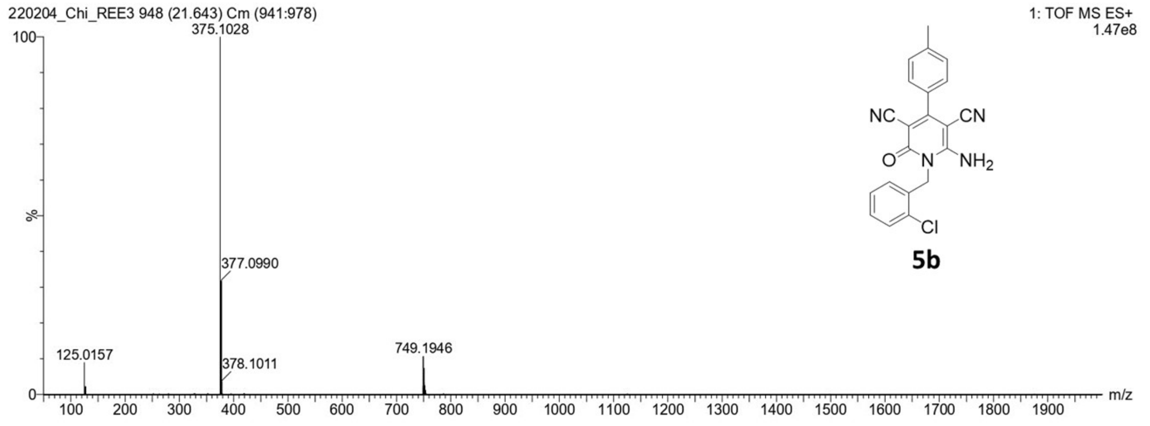

Figure S48. HRMS of compound **5b**.

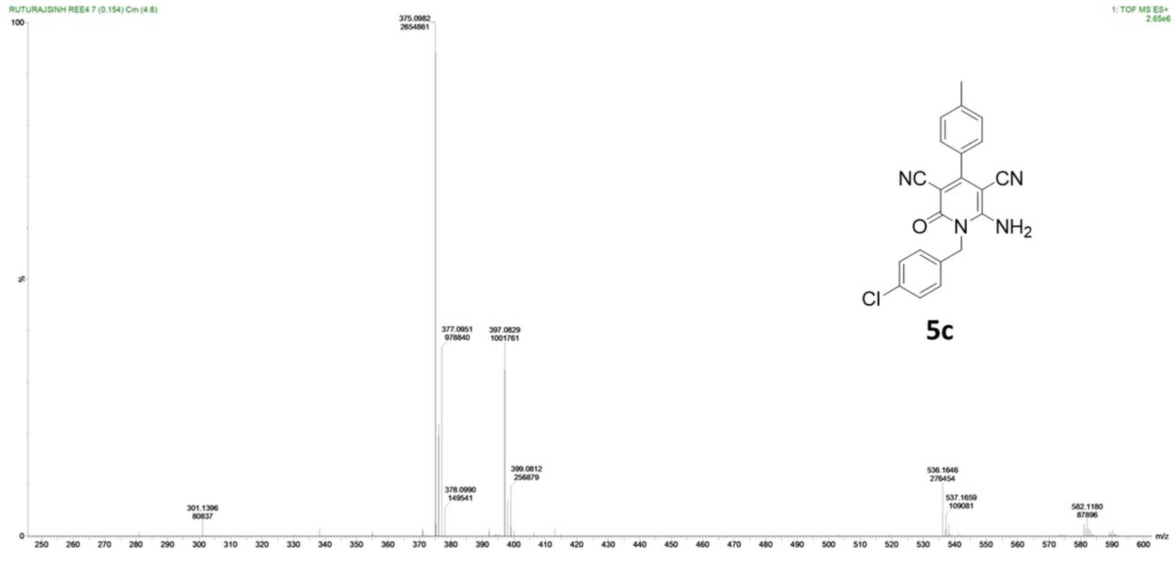

Figure S49. HRMS of compound **5c**.

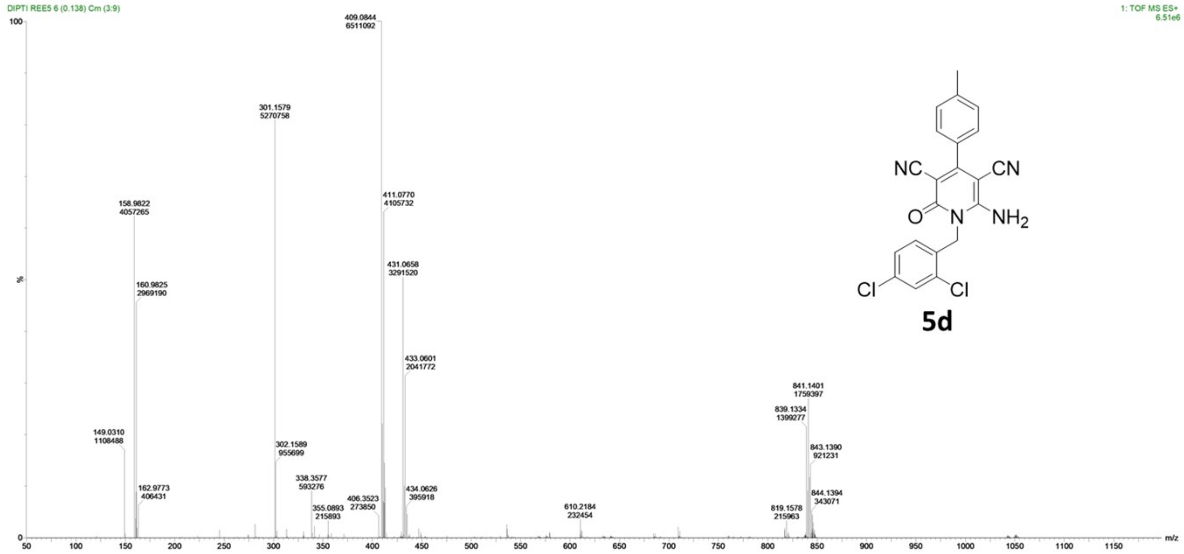

Figure S50. HRMS of compound **5d**.

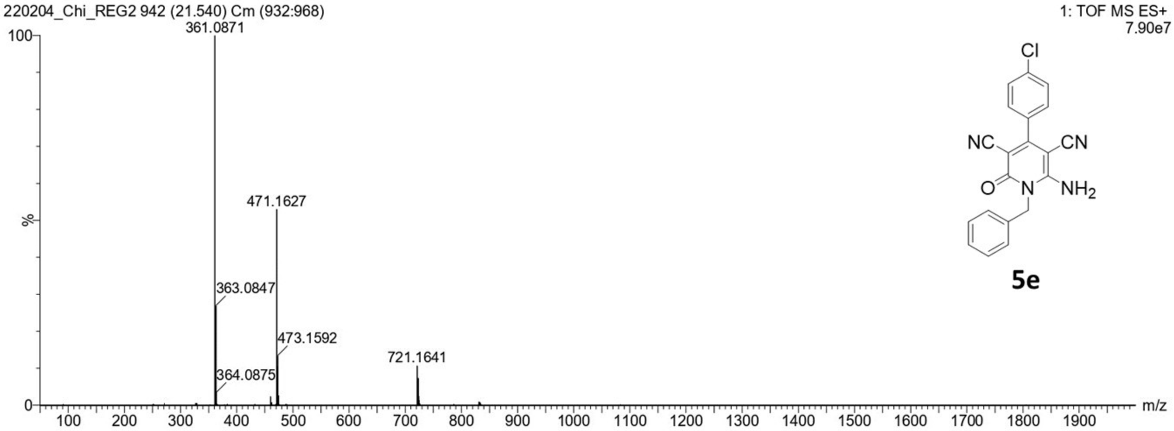

Figure S51. HRMS of compound **5e**.

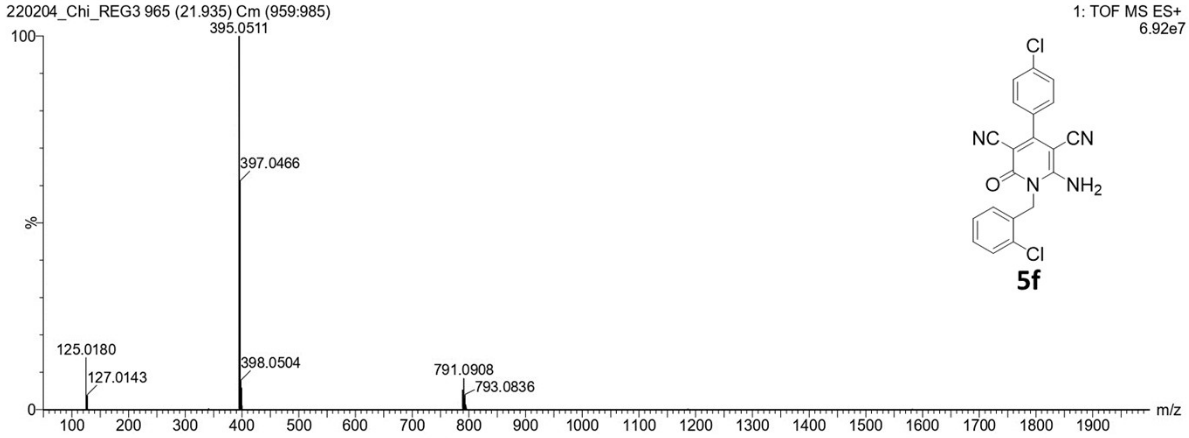

Figure S52. HRMS of compound **5f**.

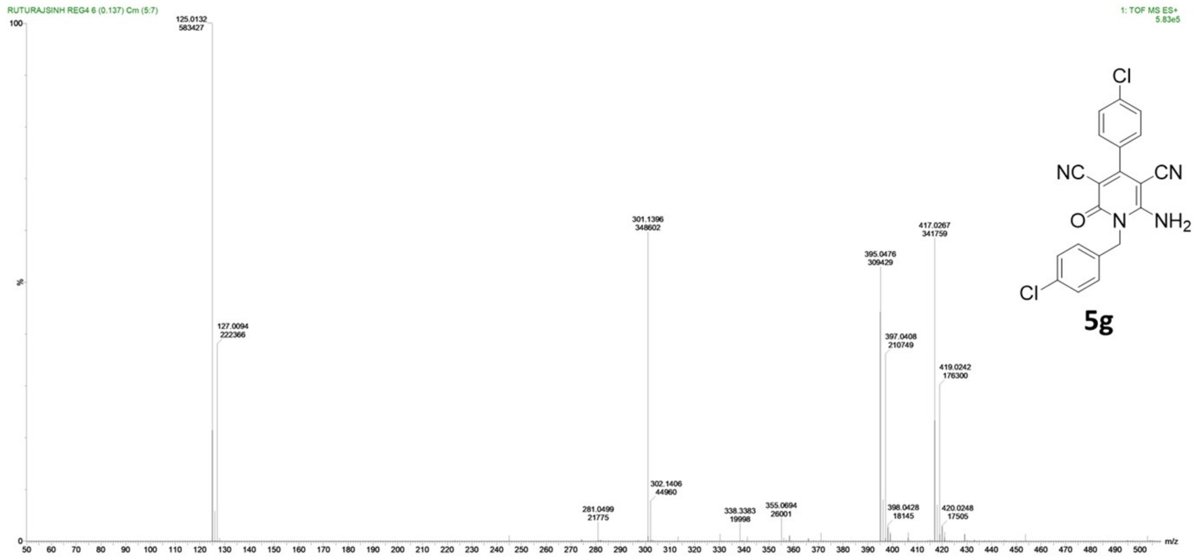

Figure S53. HRMS of compound **5g**.

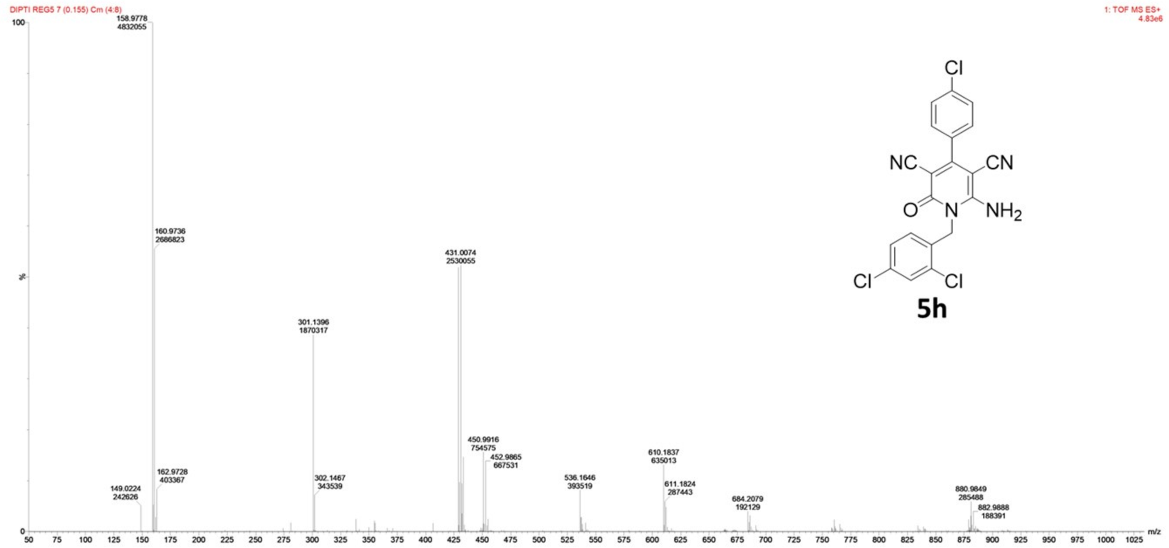

Figure S54. HRMS of compound **5h**.

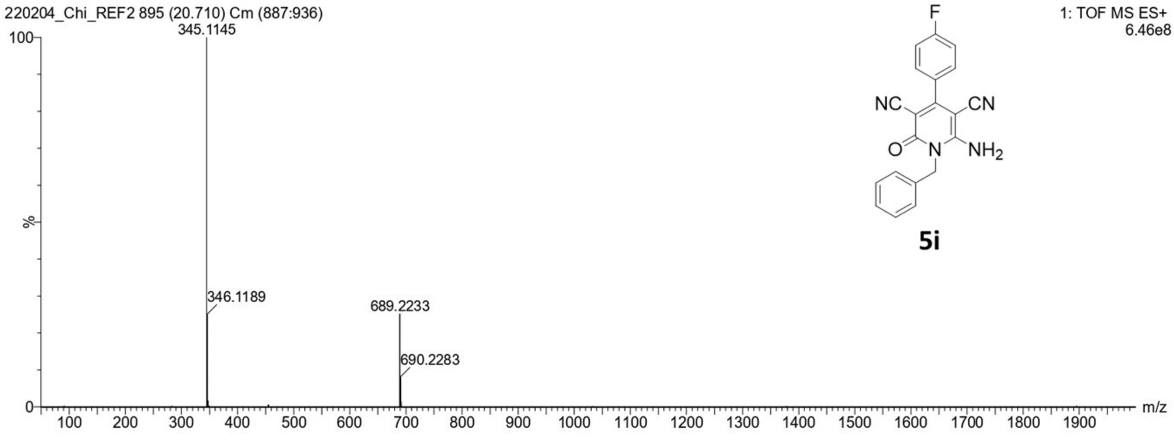

Figure S55. HRMS of compound **5i**.

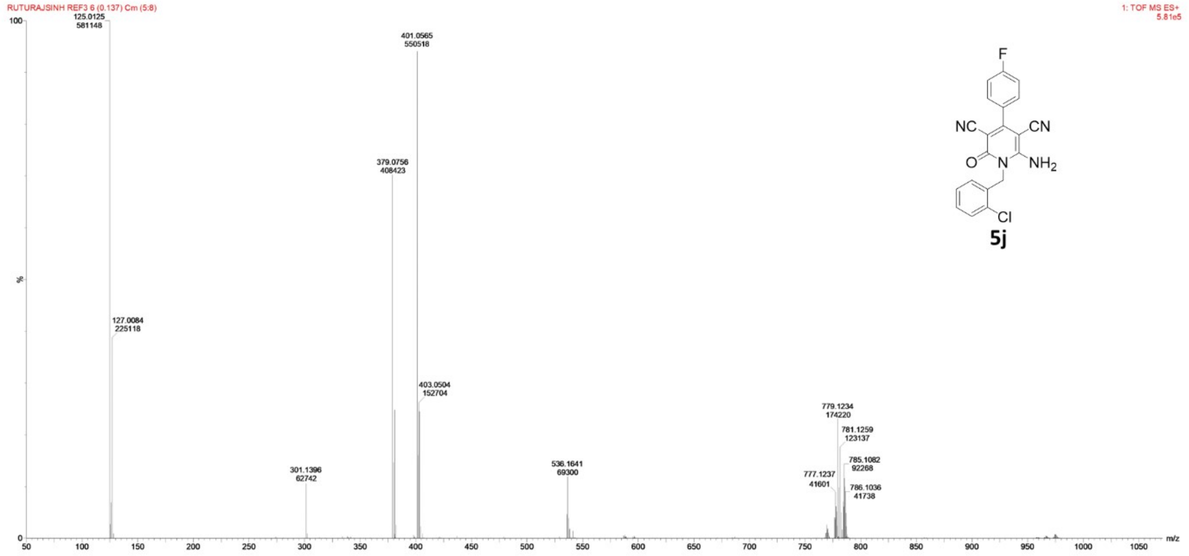

Figure S56. HRMS of compound **5j**.

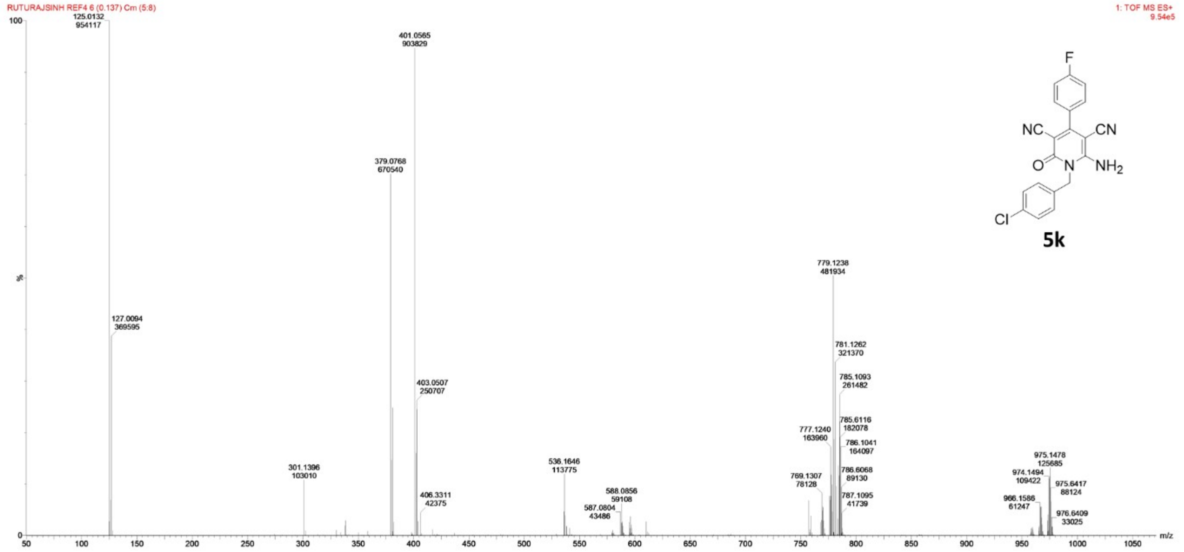

Figure S57. HRMS of compound **5k**.

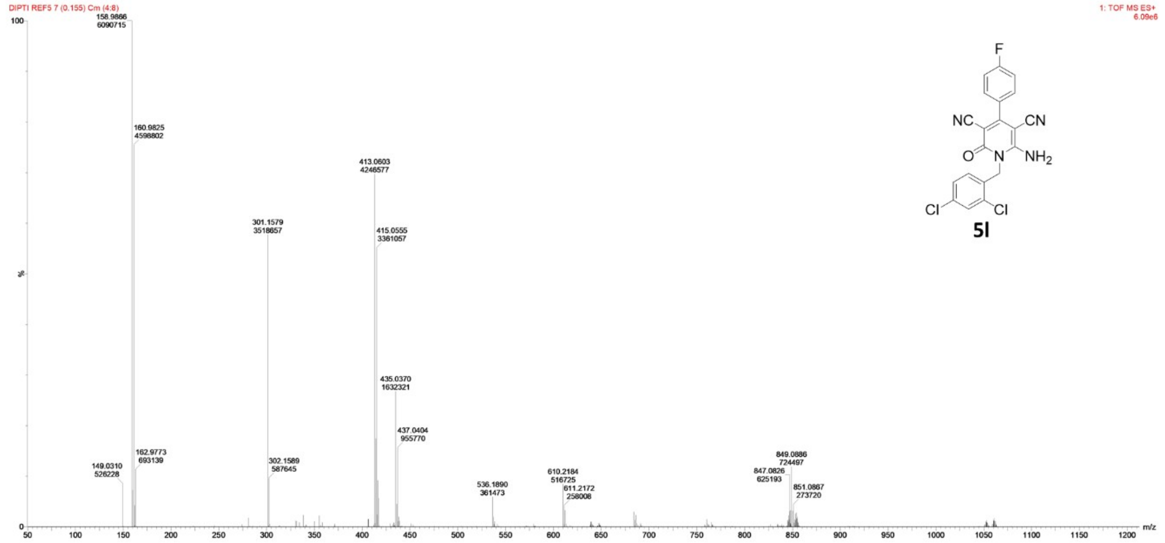

Figure S58. HRMS of compound **5l**.

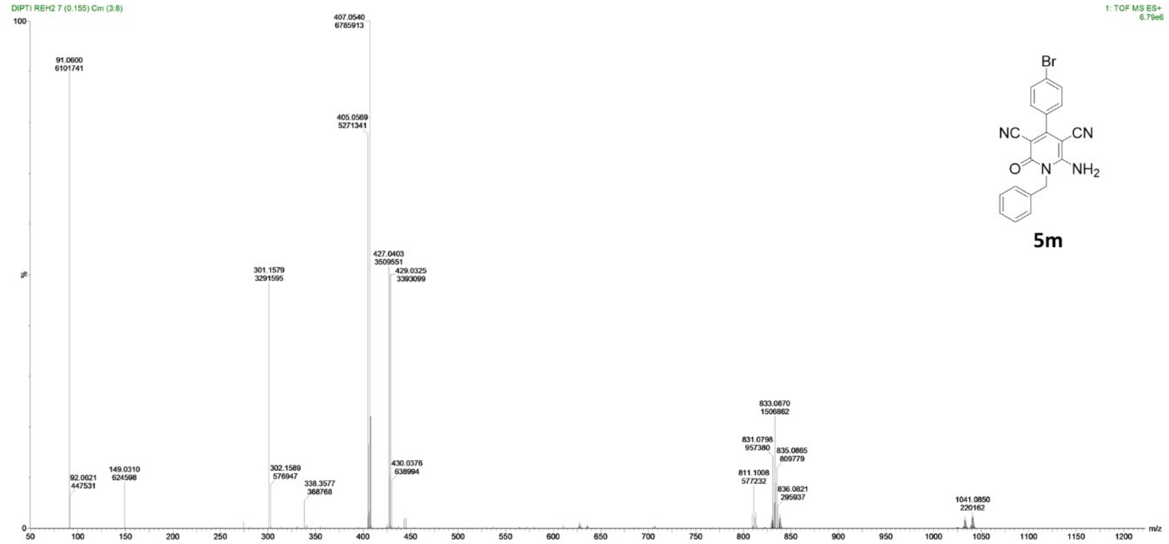

Figure S59. HRMS of compound **5m**.

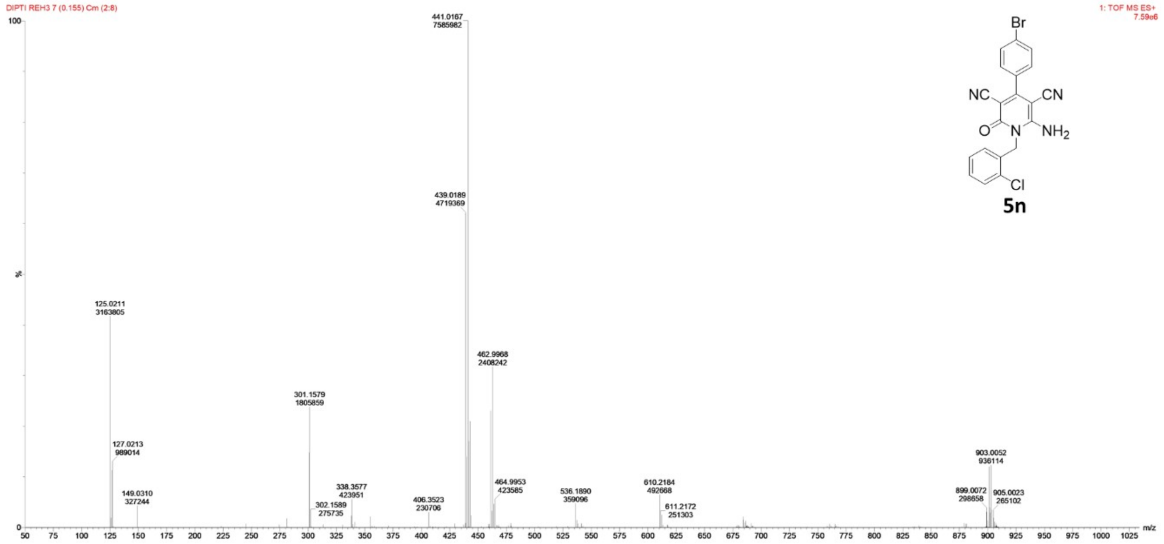

Figure S60. HRMS of compound **5n**.

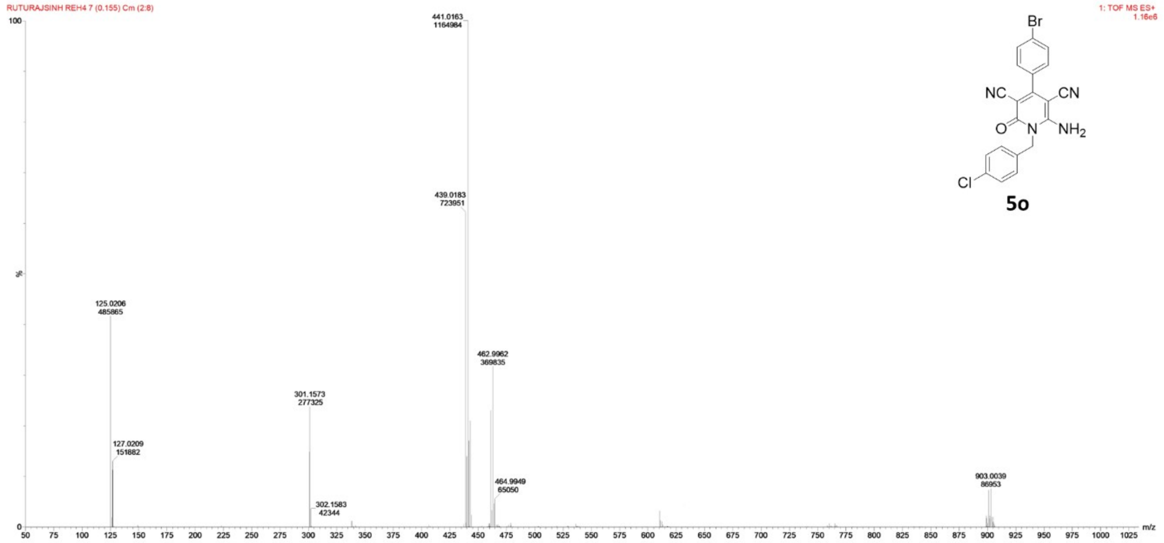

Figure S61. HRMS of compound **5o**.

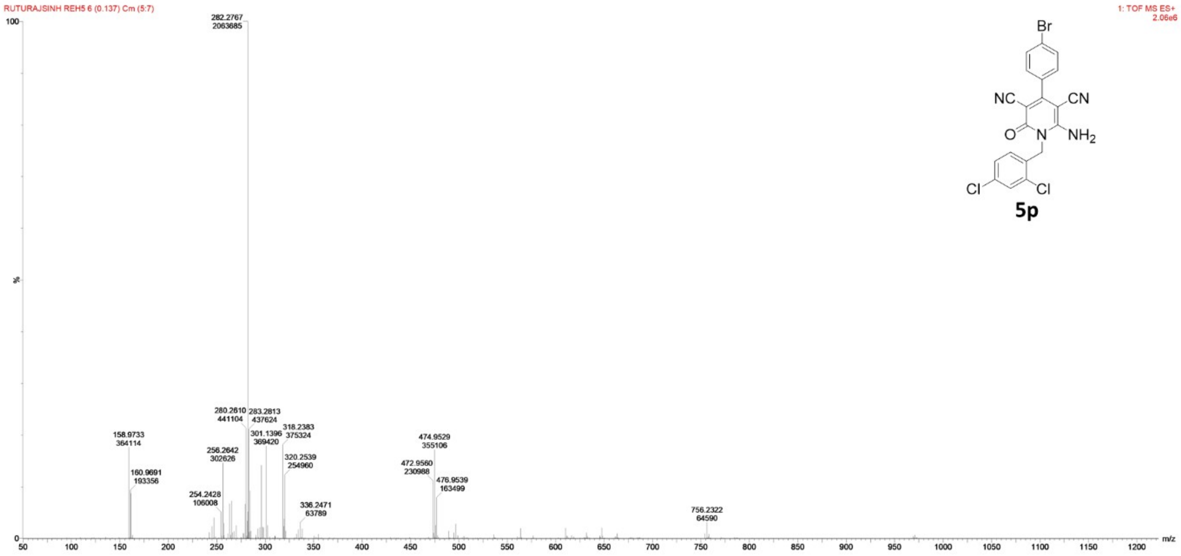

Figure S62. HRMS of compound **5p**.
